# Synthesis and properties of *N*-(trifluoromethyl)amides

**DOI:** 10.1039/d5sc08413j

**Published:** 2026-07-30

**Authors:** Miriam L. O'Duill, Isabel L. Wood, Isaac Brooks, Charlotte McIvor

**Affiliations:** a School of Chemistry, University of Nottingham, University Park Nottingham NG7 2RD UK miriam.oduill@nottingham.ac.uk

## Abstract

*N*-Trifluoromethylated amides combine the properties of two of the most important functional groups in medicinal chemistry: amides (found in >40% of all bioactive molecules) and fluorinated functional groups (found in in 23% of drugs). Therefore, it is not surprising that a growing number of publications are investigating potential *N*-(trifluoromethyl)amide drug targets. While the construction of amide bonds is the most common transformation in medicinal chemistry, the synthesis of *N*-(trifluoromethyl)amides has been a lot less straightforward until very recently. This review provides a critical overview of strategies for the synthesis of N–CF_3_ amides, highlighting recent advances, remaining challenges and promising avenues for future developments. A summary of the physical properties of N–CF_3_ amides is provided and their suitability as drug targets is evaluated.

## Abstract 2

N–CF_3_ amides are an emerging class of functional groups in medicinal chemistry. This review summarises their properties, evaluates their suitability as drug targets, and critically discusses existing strategies for their synthesis.

## Graphical abstract

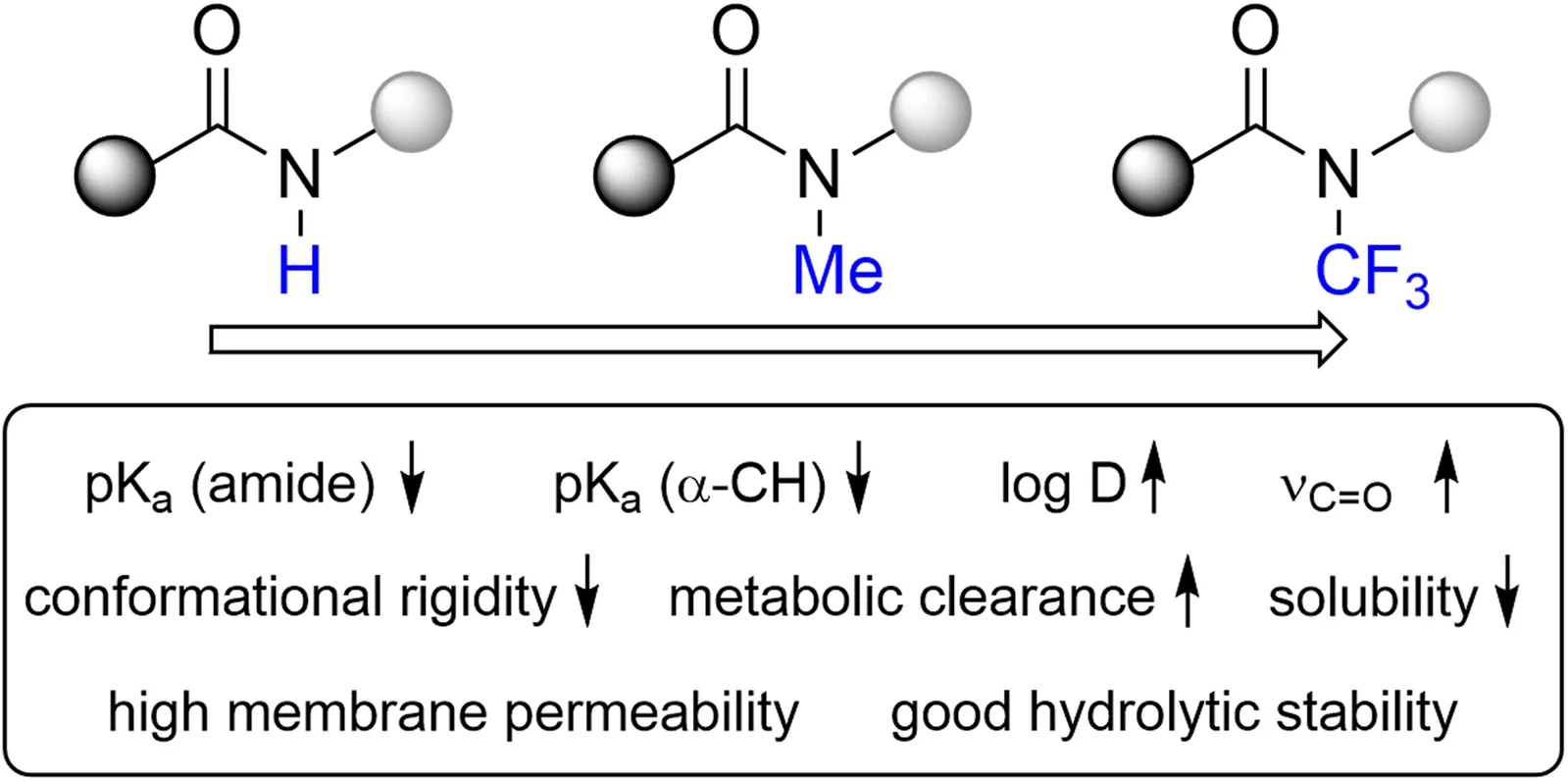

## Introduction

Amides are one of the most ubiquitous functional groups in nature.^1^ They also enjoy significant attention in medicinal chemistry, due to their unique properties as tuneable hydrogen bond donors and acceptors, as well as the stability and conformational rigidity of the amide bond.^2–4^ A 2020 analysis showed that over 40% of all bioactive molecules and drug compounds contain at least one amide moiety, followed closely by ethers (38%), tertiary amines (30%) and fluorinated functional groups (23%).^5^ The latter are routinely used to fine-tune the pharmacokinetic properties of drug targets.^6–10^ The trifluoromethyl group, in particular, has been shown to modulate p*K*_a_, lipophilicity, metabolic stability, and conformation of small molecule drugs, which in turn can have profound effects on their potency and selectivity.^11,12^

*N*-(Trifluoromethyl)amides 1 combine the advantages of both of these important functional groups, so it is not surprising that they have recently received significant attention in the literature, with a growing number of publications investigating potential *N*-(trifluoromethyl)amide drug targets (Fig. 1).^13–16^

**Fig. 1:**
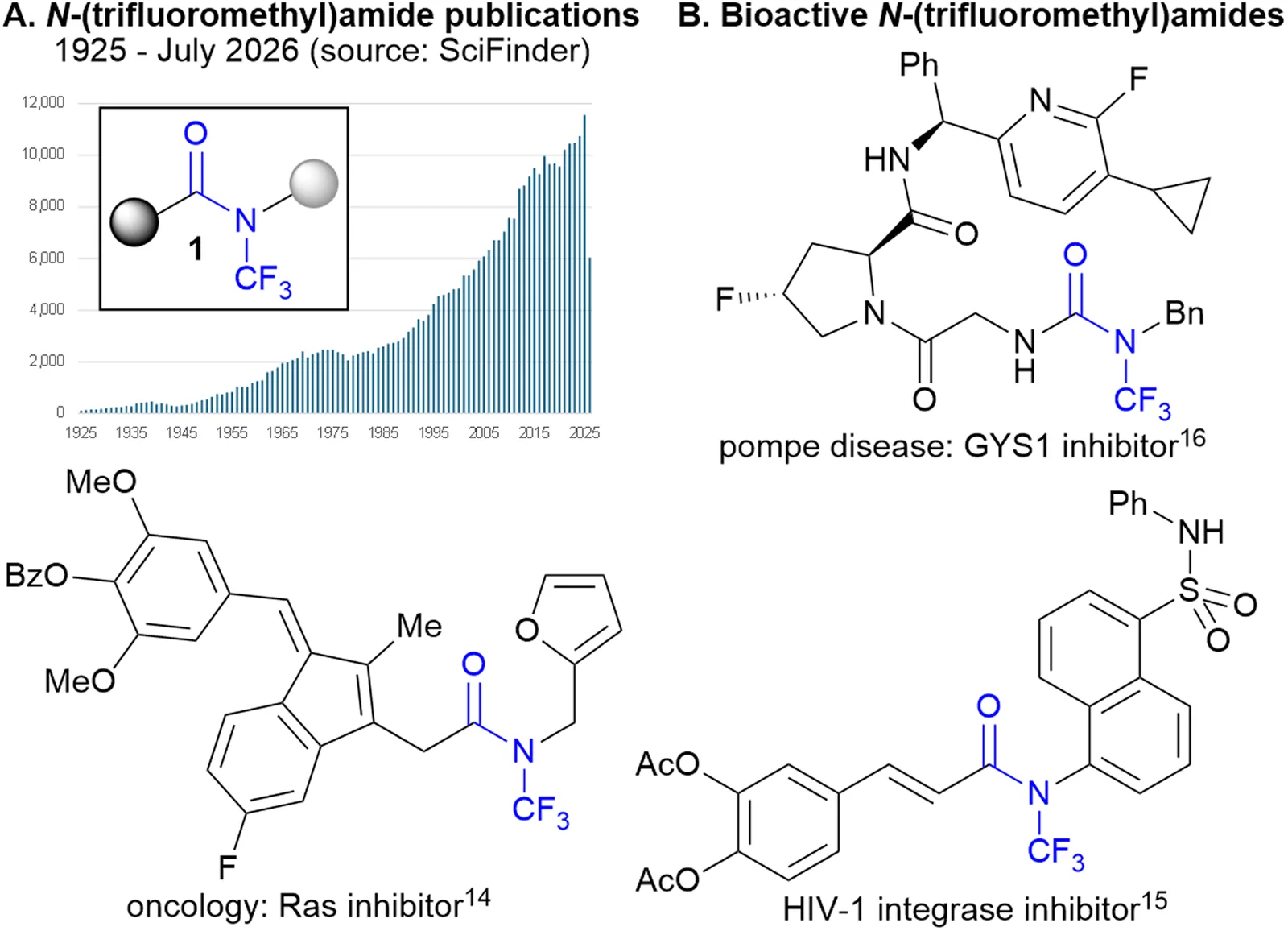
*N*-(Trifluoromethyl)amides in synthetic and medicinal chemistry.^14–17^

## Properties of *N*-(trifluoromethyl)amides

While the construction of amide bonds is the most common transformation in medicinal chemistry (representing 16% of all reactions carried out in the synthesis of new drugs),^3^ the synthesis of *N*-(trifluoromethyl)amides has been a lot less straightforward until very recently.^18–20^ Because of this, experimental data demonstrating their properties is still limited. Recent studies by Schoenebeck, Beier, Wang and Li, and Lu and Shen provide preliminary insights,^17,21–24^ but additional studies would be highly valuable for chemists working in this area.

Calculated aqueous p*K*_a_ values show that *N*-trifluoromethylation increases the acidity of the amide proton (2, Table 1). Experimentally, this trend is reflected by a substantial ^1^H NMR downfield shift compared with analogous N–H and N–Me amides, as well as an increased hydrogen bond donor strength.^22^ The acidity of the methylene protons on either side of the *N*-(trifluoromethyl)amide functional group is also increased (5).^25^

**Table 1:** Physical properties of *N*-(trifluoromethyl)amides I

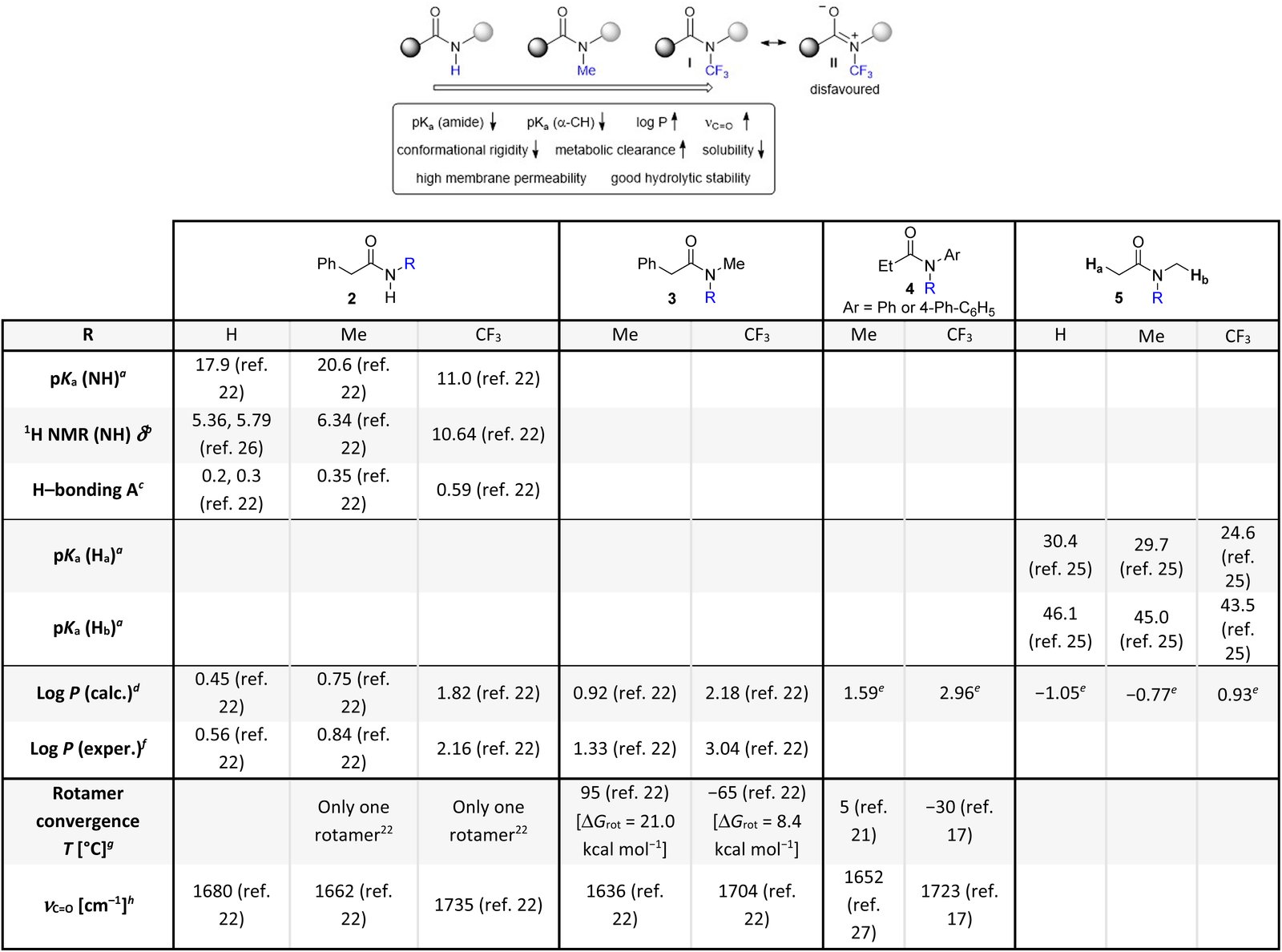

a Calculated aqueous p*K*_a_ values: BP86/TZVPD or G3MP2+/BMK level of theory with COSMO/CPCM solvation model.^22,25^

b ^1^H NMR shifts (400 MHz, CDCl_3_).^22,26^

c Hydrogen bond donor strength a, determined by ^1^H NMR.^22^

d Calculated log *P* values: COSMOTherm/StarDrop ADME QSAR.^22^

e log *P* values calculated for this review using SwissADME, XLOGP3.

f Determined using UPLC.^22^

g Determined by variable temperature ^1^H NMR.^17,22^

h Experimental IR *ν*_C=O_ vibrations.^17,22,27^

Unsurprisingly, *N*-trifluoromethylation also increases lipophilicty (log *P*/log *D*) of amides when compared with their N–H and N–Me analogues both computationally^17^ and experimentally (2–5, Table 1; 19, 28–30, Table 3).^22–24^ Log *D*_7.4_ values measured for N–CF_3_ amides were comparable to their *N*-isopropyl analogues.^24^

Variable temperature NMR studies observed only one rotamer for secondary amides, due to the strong thermodynamic preference (2–3 kcal mol^−1^) for the amide bond to adopt a *trans* conformation (2, Table 1).^22^ For tertiary amides 3 and 4, the rotamer convergence temperature and thus the barrier to rotation around the amide bond was found to be lower in N–CF_3_ amides than their N–Me analogues.^17,22^ This suggests that introduction of the electron-withdrawing CF_3_ group reduces conjugation of the nitrogen lone pair (Table 1, resonance structure II), decreasing conformational rigidity. This trend is also reflected by a blue shift of the *ν*_C=O_ vibration in the IR spectrum and a lengthening of the C(sp^2^)–N bond in XRD structures,^28–30^ indicating a stronger C=O bond and reduced C–N double bond character, respectively.^17,22^

Despite this weakening of the amide bond, *N*-(trifluoromethyl)-amides have shown sufficient stability towards hydrolysis for applications in drug discovery (Table 2).^17,21–24^ Most tertiary N–CF_3_ amides (6–10, 15–19) showed minimal to no decomposition in neutral aqueous solution (pH 7.4) over 6 h at room temperature or 50 °C, with half-lives of at least 10 days and often exceeding 60 days.^17,21,23,24^ Similar results were observed in human blood plasma: 67–98% of the *N*-(trifluoromethyl)amides tested (7–10, 16–19) were recovered after incubation for 18 hours at 37 °C.^24^ Under acidic conditions (pH 1), most N–CF_3_ amides exhibited good stability, but structural effects became more pronounced: *N-*(aryl)aryl amides (6–7) retained half-lives of >60 days,^17,21,23,24^ while some *N*-(aryl)alkyl (9–10), *N*-(alkyl)aryl (16) and *N*-(alkyl)alkyl amides (18–19) showed shorter half-lives of 5–22 days.^22,24^ In these cases, structural modification such as cyclopropylation of the carbonyl moiety (8, 17) restored excellent hydrolytic stability.^24^ Under basic conditions (pH 10), the majority of N–CF_3_ amides were highly labile with half-lives of only 1–6 days (7–10, 16–19, Table 2).^17,21,23,24^

**Table 2:** Hydrolytic stability of *N*-(trifluoromethyl)amides

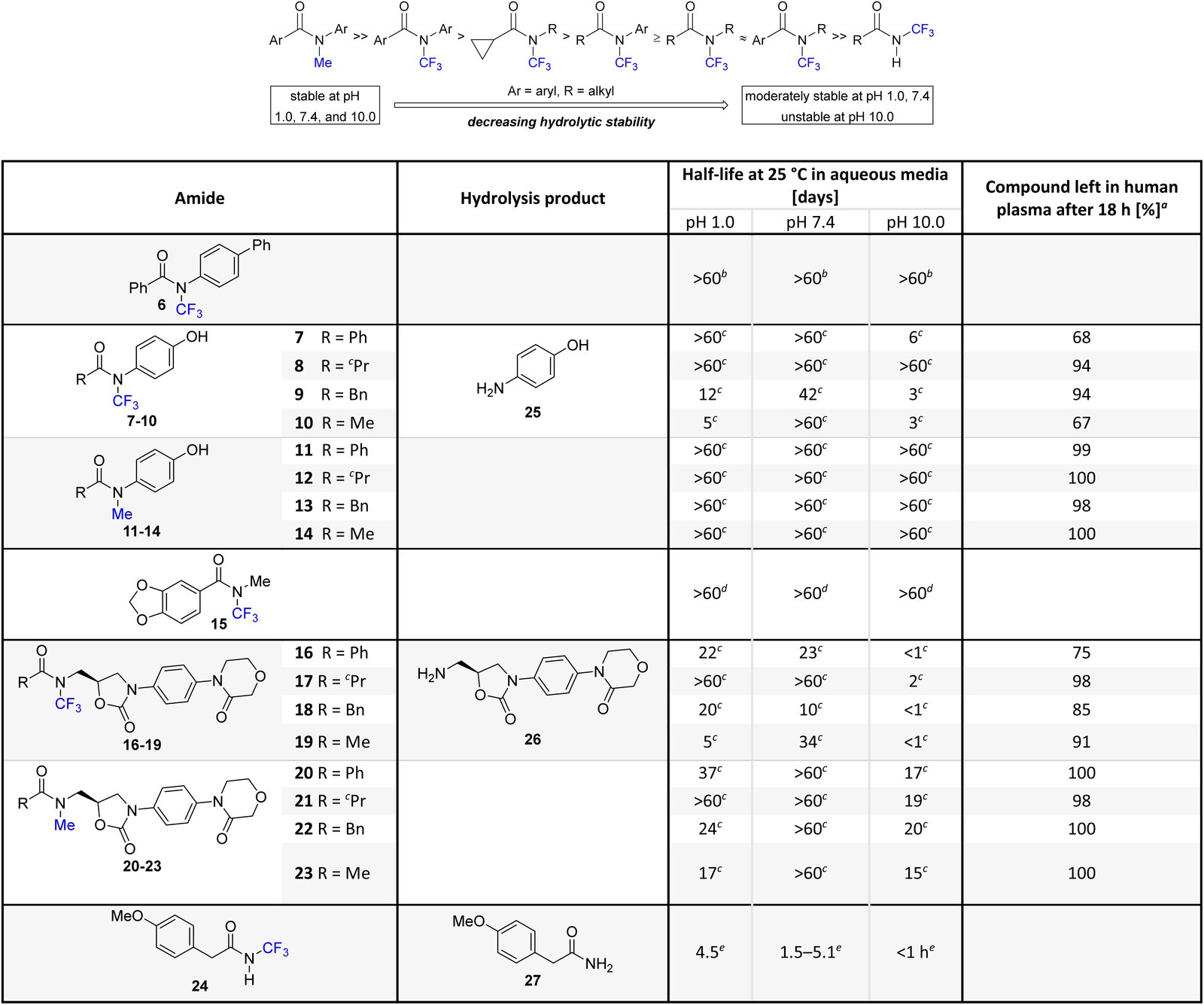

a Amount of compound left in human plasma after 18 h at 37 C.^24^

b No decomposition observed in water/DMSO (4 : 1) at pH 1.0, 7.4 and 10.0 after 6 h.^21^

c Determined in 0.1 M HCl solution, 20 mM sodium phosphate buffer, and 20 mM sodium carbonate buffer, respectively.^24^

d Determined in aqueous HCl solution (pH 2.0), Fetal Bovine Serum (FBS) (pH 7.4) and sodium carbonate buffer (SCB) (pH 9.6), respectively.^23^

e Determined in MeCN/1M aq. HCl, MeCN/water (1 : 1 and 9 : 1), and MeCN/1M aq. NaOH, respectively.^22^

Secondary N–CF_3_ amides were substantially less stable towards hydrolysis than tertiary N–CF_3_ amides. Amide 24 showed half-lives of 1.5 and 5 days in MeCN/water solutions (1 : 1 and 9 : 1, respectively),^22^ and hydrolysed fully within 1 hour at room temperature under alkaline conditions (Table 2).^22^

Comparisons with non-trifluoromethylated amides are contradictory and may be highly structure-dependent: while N–CF_3_ amide 4 (Table 1) was reported to be more stable at pH 10 than its N–Me and N–H analogues,^17^ the most recent large-scale study showed substantially higher stability of N–Me amides both in aqueous solution and in human blood plasma (11–14, 20–23, Table 2).^24^

Where hydrolysis occurred, the main decomposition product for tertiary N–CF_3_ amides was the primary amine (25, 26),^24^ formed by hydrolysis of both the amide and the trifluoromethyl moieties, while secondary N–CF_3_ amides gave the primary amide (27, Table 2).^22^

*In vitro* metabolic stability of *N*-(trifluoromethyl)amides was evaluated in human liver microsomes (HLM).^24^ Intrinsic clearance (CL_int_) values were in line with lipophilicity trends: N–CF_3_ amides cleared faster than their N–Me and N–H analogues (Table 3). When cytochrome P450-mediated oxidation was suppressed to specifically investigate the stability of N–CF_3_ amides towards enzymatic hydrolysis (HLM-N), *N*-(alkyl)(trifluormethyl)amides (28) showed comparable stability to their non-trifluoromethylated analogues, while *N*-(aryl)(trifluoromethyl) amides (29, 30) were less stable (Table 3).^24^ The main metabolite observed in these experiments was the carboxylic acid resulting from amide hydrolysis.^24^

**Table 3:** Physical properties of *N*-(trifluoromethyl)amides II^24^

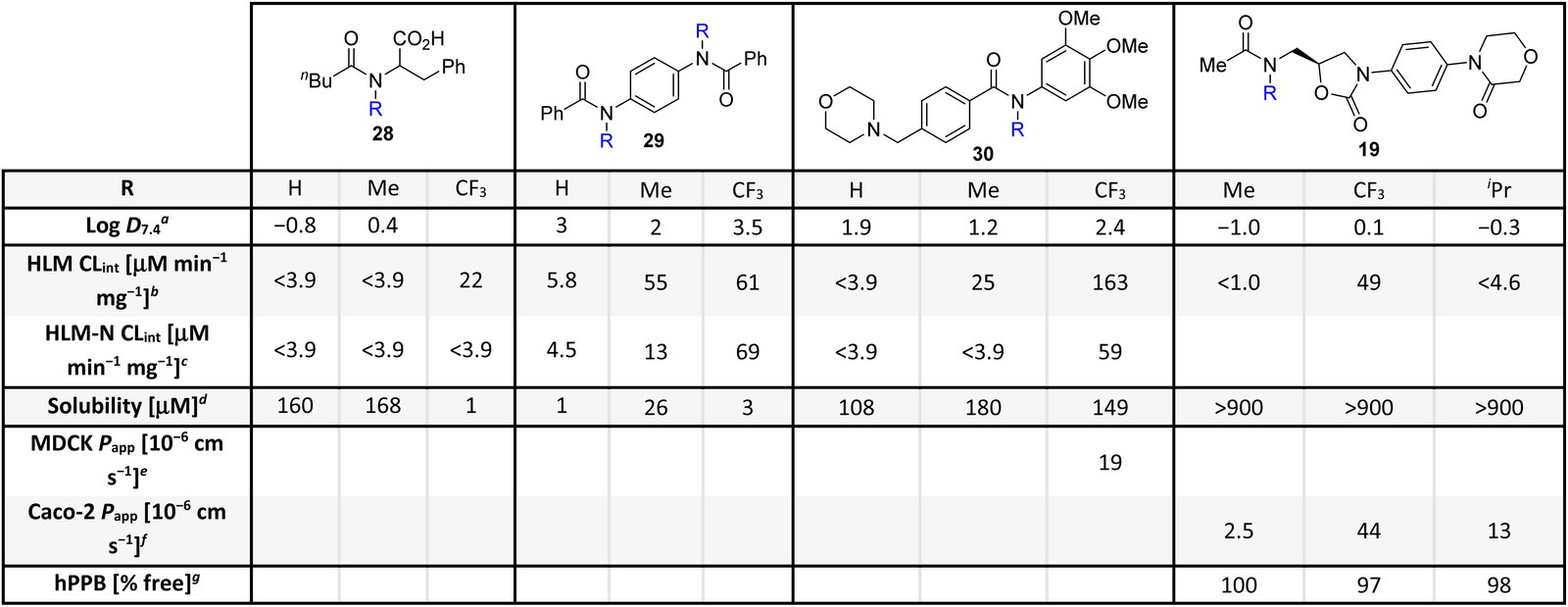

a Experimental log *D*_7.4_ measurements determined by microscale shake-flask method.

b Metabolic stability: intrinsic clearance values, measured in human liver microsomes with NADPH.

c Metabolic stability: intrinsic clearance values, measured in human liver microsomes without NADPH.

d Kinetic solubility of the compounds as DMSO stock solutions (10 mM) measured in PBS buffer (pH = 7.4) at a final concentration of 200 µM after shaking for 24 h at rt.

e Membrane permeability in MDCK cells with endogenous canine MDR1 genetically ablated.

f Apical-to-basolateral passive permeability across the Caco-2 cell monolayer in the presence of inhibitors against the three major efflux transporters: P-glycoprotein (P-gp), breast cancer resistance protein (BCRP), and multidrug-associated protein 2 (MRP2).

g Human plasma protein binding: unbound compound remaining after shaking of DMSO stock solutions of the compound in human plasma (final concentration 7 µM) for 18 h.

Solubility, membrane permeability and human plasma binding of N–CF_3_ amides are also in line with lipophilicity trends. *N*-(Trifluoromethyl)amides showed lower kinetic solubility in Phosphate-Buffered Saline (PBS) at pH 7.4 than their N–Me and N–H analogues (28–30, Table 3), although solubility could be improved by adding solubilising functional groups such as morpholine (19, 30) or hydroxyazetidine.^24^ High membrane permeability was observed in Madin–Darby canine kidney (MDCK) and human epithelial colorectal adenocarcinoma (Caco-2) cells (19, 30, Table 3). Minimal binding of N–CF_3_ amides to human plasma protein was observed (97% unbound compound 19 was recovered after 18 hours), ensuring a large proportion of “free” drug available for therapeutic action. *In vivo* pharmacokinetic data of *N*-(trifluoromethyl)amides has not yet been obtained at the time of writing, but N–CF_3_ ureas showed a similar pharmacokinetic profile in rats to their N–Me analogues.^24^

Preliminary data on the physiochemical and *in vitro* pharmacokinetic properties of *N*-(trifluoromethyl)amides suggests that they are suitable targets for drug discovery programmes. While less stable than C–CF_3_ compounds, their hydrolytic stability is generally sufficient for most pharmaceutical applications and can be controlled through structural modifications. This data shows promise for the application of N–CF_3_ amides in medicinal chemistry, but the current dataset is limited, and additional studies would be highly valuable for practitioners in this field.

## Methods for the synthesis of *N*-(trifluoromethyl)amides

### Scope of this review

The two main approaches for the synthesis of *N*-(alkyl)amides, amide coupling and electrophilic *N*-alkylation, cannot be directly translated to the synthesis of N–CF_3_ amides. This review aims to provide an overview of alternatives developed for the synthesis of *N*-(trifluoromethyl)amides (Fig. 2):

**Fig. 2:**
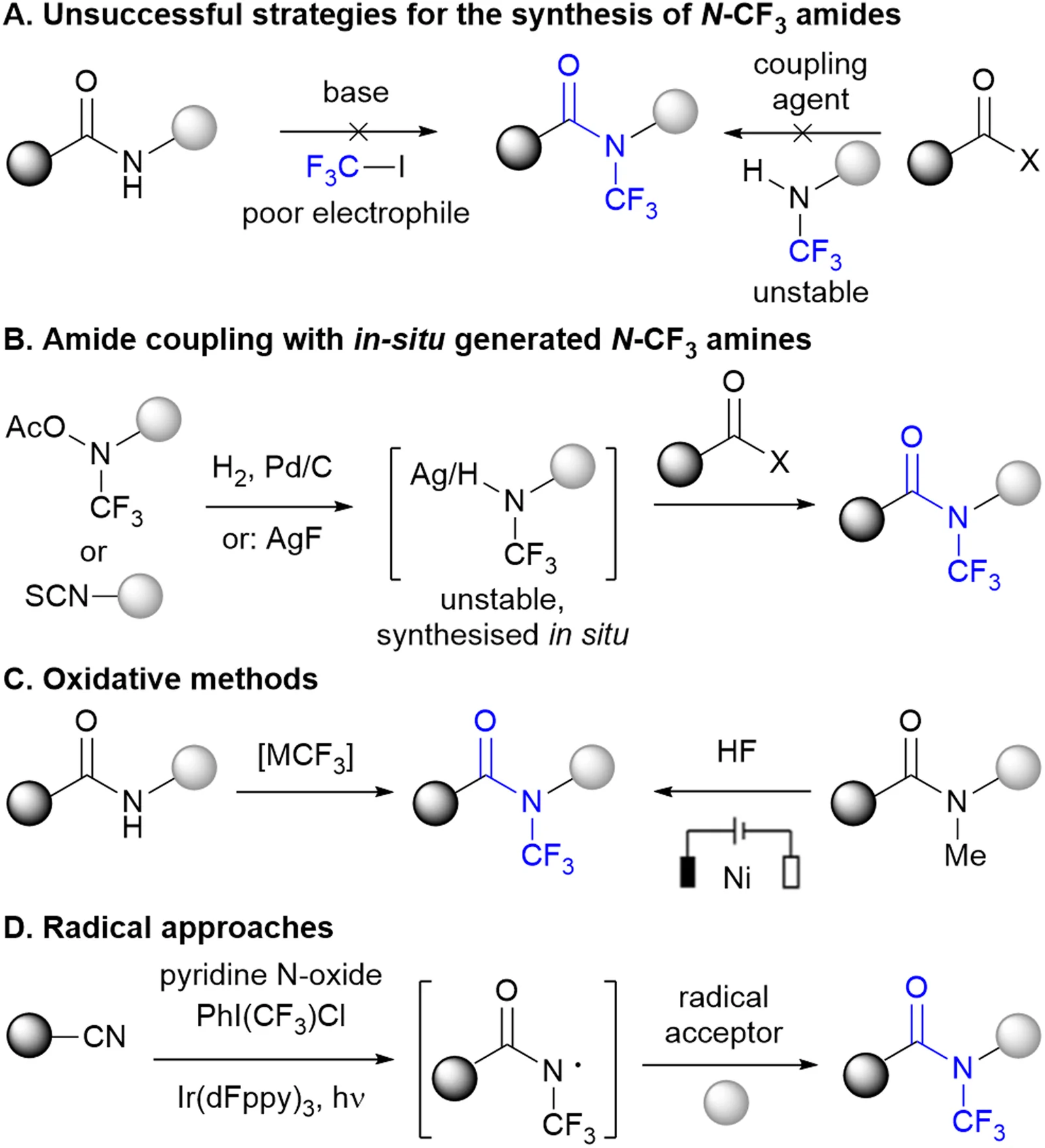
Scope of this review: synthesis of *N*-(trifluoromethyl)amides.

Amide coupling reactions to access N–CF_3_ amides are challenging due to the poor nucleophilicity of *N*-(trifluoromethyl)amines and their propensity to undergo fluoride elimination and hydrolysis (Fig. 2A). To circumvent this issue, methods for the *in situ* formation of N–CF_3_ amines from N–CF_3_ hydroxylamines, or metal-stabilised N–CF_3_ azanide anions from isothiocyanates will be discussed (Fig. 2B). Electrophilic trifluoromethyl sources such as trifluoroiodomethane also show low reactivity (Fig. 2A), requiring oxidative alternatives which enable trifluoromethylation of secondary amides with [MCF_3_] (M = Ag, Cu) or direct C–H fluorination of *N*-(methyl)amides under electrochemical conditions (Fig. 2C). Finally, a radical approach has recently been reported in which nitriles are used as a source of N–CF_3_ amidyl radicals which show improved stability compared with N–CF_3_ amines (Fig. 2D).

### Early methods using gaseous reagents

Early methods for the synthesis of *N*-(trifluoromethyl)amides used gaseous N–CF_3_ reagents whose low boiling points (ranging from −83 °C to +18 °C), hazardous synthesis, and high reactivity made them impractical for general use (Fig. 3).^31,32^ While the yields reported in these transformations were often good,^33–42^ functional group compatibility was low and most of the products were structurally very simple.

**Fig. 3:**
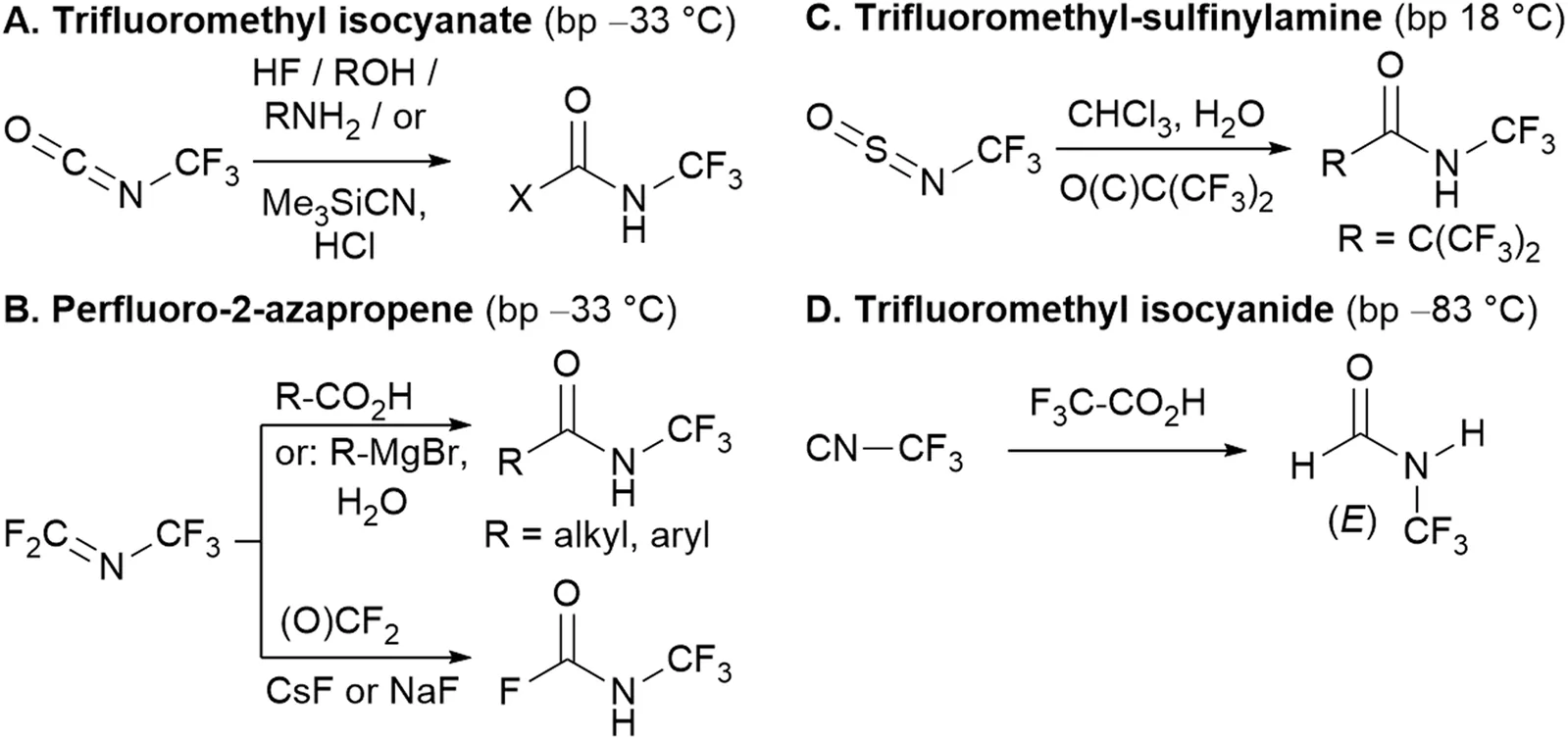
Early methods for the synthesis of *N*-(trifluoromethyl)amides using gaseous reagents.^33–42^

Formation of these reagents *in situ* allows for their use in a safer, more practical setting. Trifluoromethyl isocyanate, for example, can be generated from trifluoroacetic acid and trimethylsilyl azide through a Curtius rearrangement, allowing access to the steroidal N–CF_3_ tetrazolone 31, though the functional complexity of this molecule is still very limited and no yields were reported (Scheme 1).^43^

**Scheme 1:**
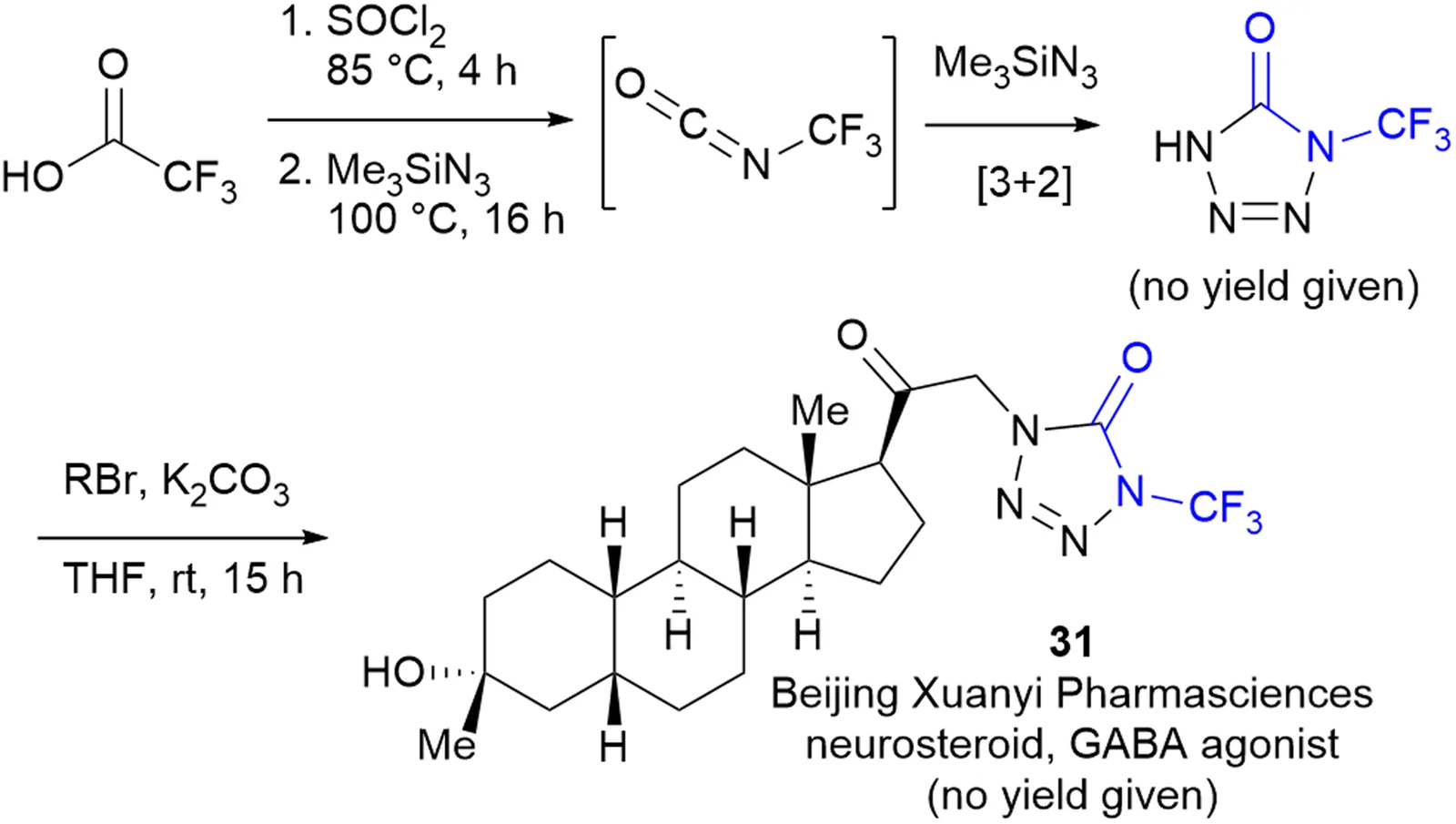
*In situ* formation of trifluoromethyl isocyanate in the synthesis of *N*-trifluoromethylated tetrazolones.^43^

### Amide coupling with *N*-(trifluoromethyl)amines

The most common strategy for amide bond formation is reaction of carboxylic acid derivatives with amines.^1,44^ The synthetic chemist attempting to use this strategy for the synthesis of *N*-(trifluoromethyl)amides, however, will experience a series of challenges (Fig. 4): primary and secondary trifluoromethylamines 32 are difficult to synthesise,^15,42,45–51^ and are prone to decomposition by fluoride elimination (dehydrofluorination) or hydrolysis,^11,17,41^ potentially forming toxic and explosive products such as cyanogen fluoride. Furthermore, trifluoromethylamines are poor nucleophiles due to the steric bulk and strong electron-withdrawing effect of the CF_3_ group. As such, this strategy often fails,^52^ with only limited examples in the patent literature, most of which do not report the yield of products obtained (Fig. 5).^14,53^

**Fig. 4:**
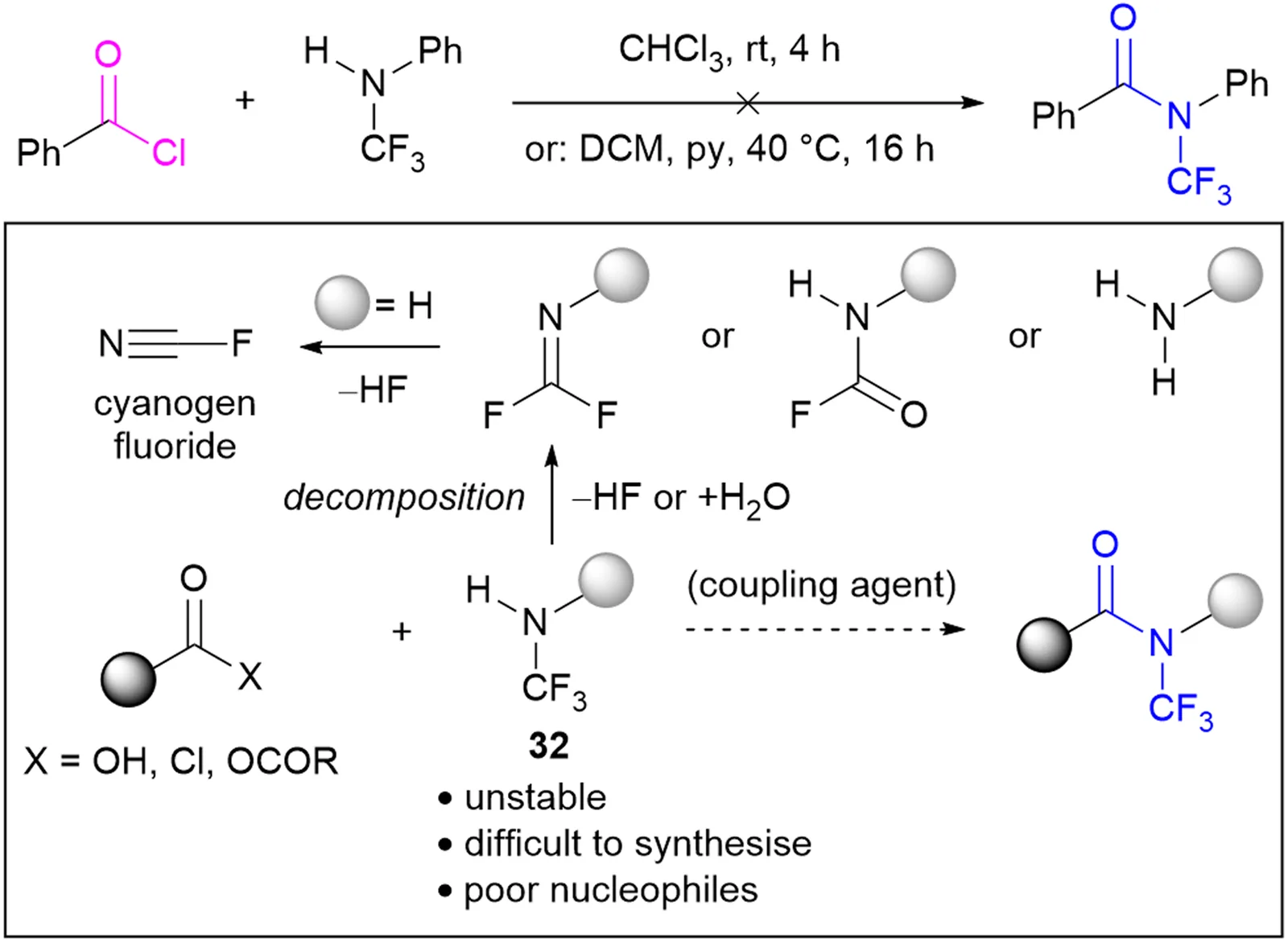
Challenges in amide coupling with *N*-(trifluoromethyl)amines.^52^

**Fig. 5:**
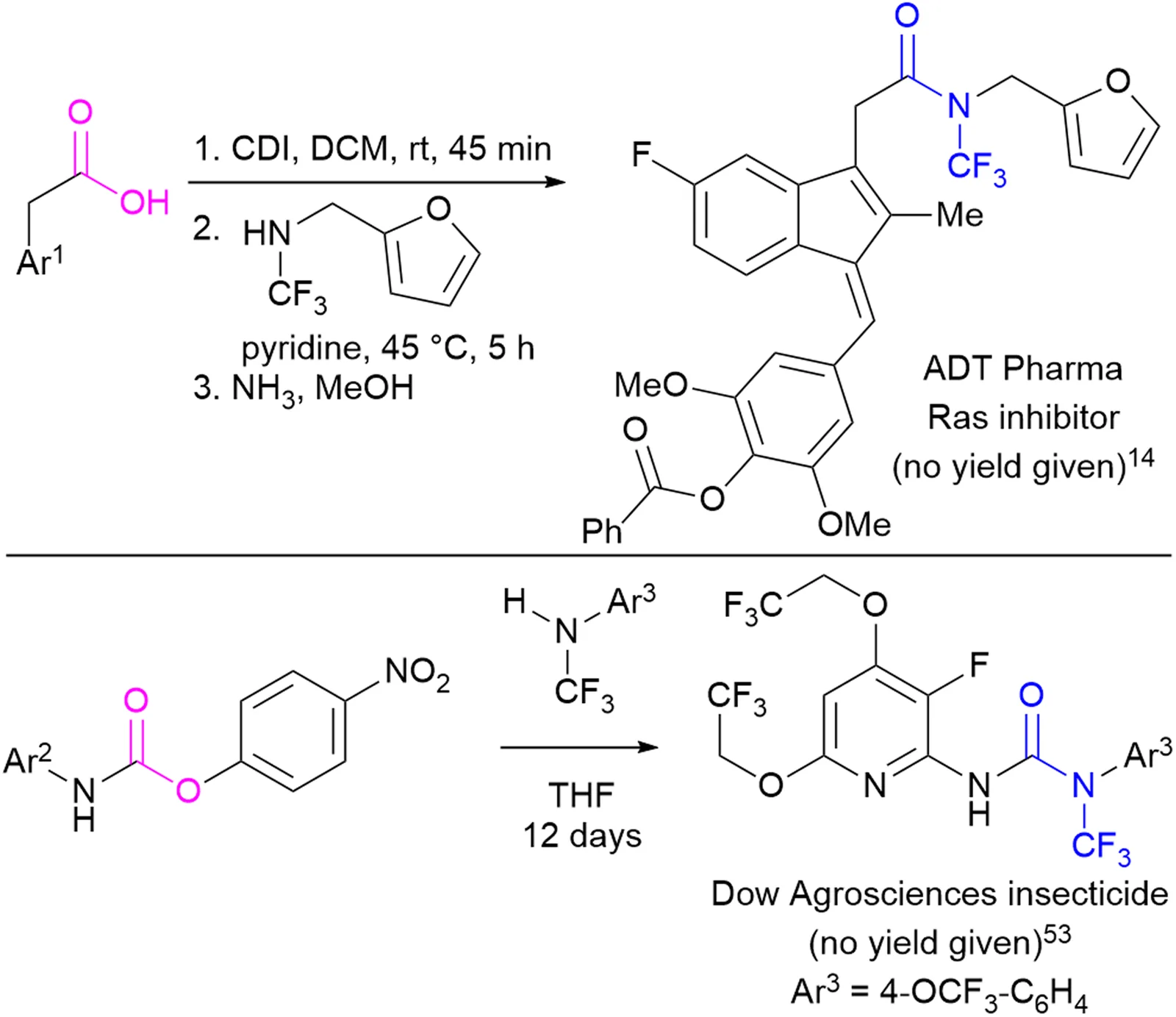
Limited examples of amide coupling with *N*-(trifluoromethyl)amines.^14,53^

To avoid decomposition by dehydrofluorination, the required *N*-(trifluoromethyl)amine can be formed *in situ* before trapping it with electrophiles.

### Isothiocyanates as *N*-(trifluoromethyl)amine precursors

Isothiocyanates have become one of the most important precursors for the *in situ* formation of *N*-(trifluoromethyl)-amines. In 1965, Sheppard discovered that treatment of isothiocyanates 33 with HgF_2_ at 200 °C resulted in desulfurisation and fluorination, forming difluoromethylimines 34 (Scheme 2).^54^ These species were difficult to handle and etched glass vessels, but could be trapped with CsF to form N–CF_3_ azanide anions 35, which showed improved nucleophilicity towards fluorophosgene compared with N–CF_3_ amines 32.

**Scheme 2:**
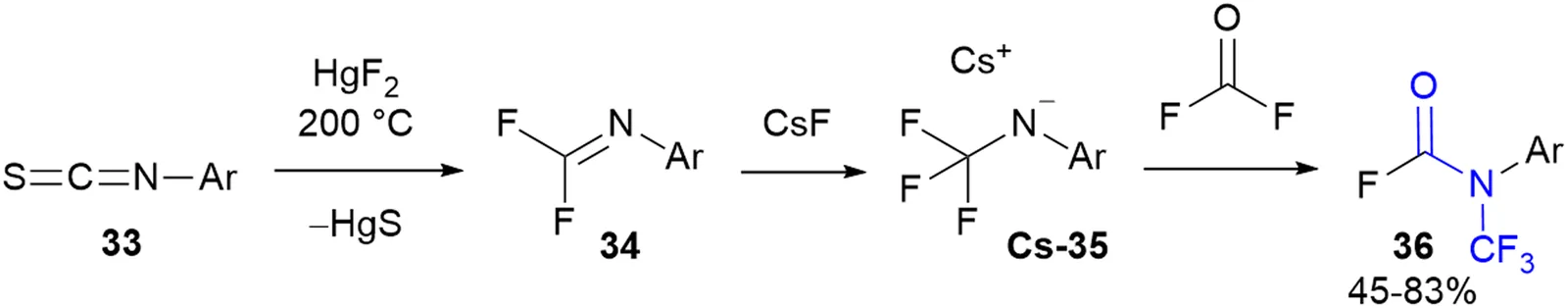
Sheppard's desulfurisation-fluorination of isothiocyanates.^54^

In 2009, Rozen *et al.* demonstrated that dithiocarbamate derivatives 37, accessed by sequential reaction of isothiocyanates 33 with ethanethiol and carboxylic acids, can complex BrF_3_ to achieve a similar desulfurization-fluorination, accessing a range of *N*-(alkyl)-*N*-(trifluoromethyl)amides (Fig. 6).^55^ Alkyl halide (39) and ester (40) functionalities were tolerated, as well as heteroarenes (42). Overall, however, the method's functional group compatibility was relatively low, given the high reactivity of BrF_3_. In some cases, fluorination of the carbonyl also occurred, but the resulting by-product 45 easily hydrolysed to the desired product (38–43) during workup for *N*-(alkyl)amides. *N*-(Aryl)amides were unsuccessful substrates under these conditions, as by-product 45 was very stable towards hydrolysis resulting in low yields of the N–CF_3_ amide (44).

**Fig. 6:**
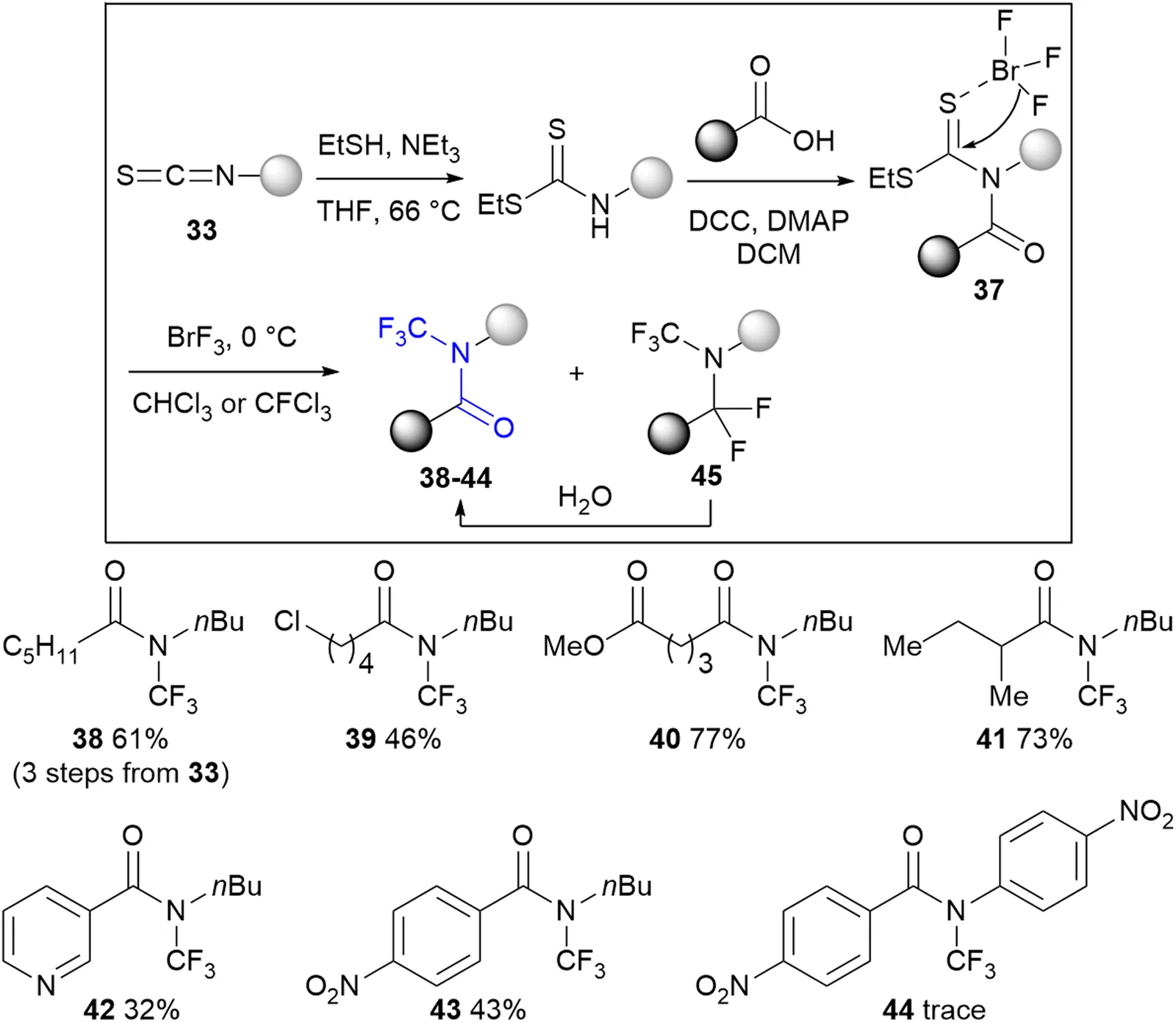
Trifluoromethylation of thiocarbamates with BrF_3_. (Yields are given over 3 steps from the corresponding isothiocyanate 33.)^55^

In 2019, Schoenebeck *et al.* achieved a breakthrough by recognising that desulfurisation-fluorination could be achieved with AgF, and that the silver countercation stabilised the N–CF_3_ azanide 35 towards hydrodefluorination.^17,56^ Using this method in the presence of triphosgene, they were able to access a wide range of versatile, bench-stable *N*-trifluoromethyl carbamoyl fluorides 36 in excellent yields and with outstanding functional group compatibility. Further functionalisation with a variety of nucleophiles allowed access to a wide range of functionally rich N–CF_3_ amide derivatives, opening up a large area of chemical space (Fig. 7).

**Fig. 7:**
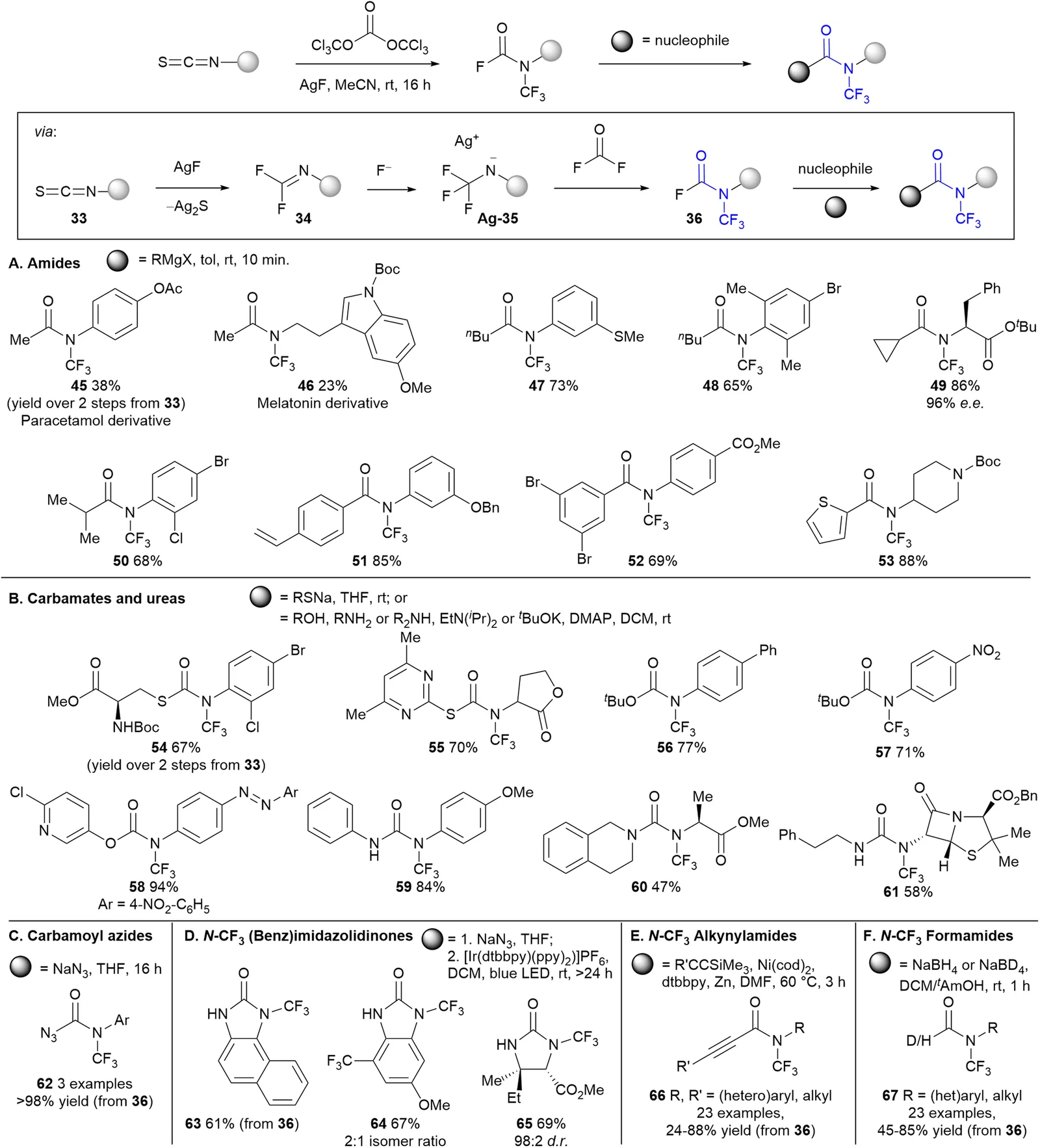
Formation of N–CF_3_ carbamoyl fluorides 36 from isothiocyanates and their derivatisation.^17,57–61^ (Yields are given over 2 steps from the corresponding isothiocyanate 33, or – where specified – directly from the corresponding carbamoyl fluoride 36).

Reaction of *N*-trifluoromethyl carbamoyl fluorides 36 with Grignard reagents afforded *N*-trifluoromethylated amides (45–53) bearing alkyl, aryl and heteroaryl substituents (Fig. 7A).^17^ Steric bulk was tolerated well (48–50), though no examples of tertiary isothiocyanides or Grignard reagents were included. A wide range of functional groups were tolerated, including tertiary amines, amides, nitriles, esters, sulfones, thioethers, halogens, nitro and diazo groups, providing valuable handles for further functionalisation. Protic groups required a protecting group in both steps. Optically pure ^*t*^Bu-protected amino acids were transformed with retention of stereochemistry (49).

Reaction of N–CF_3_ carbamoyl fluorides 36 with thiolate, selenate, alcohol and amine nucleophiles afforded seleno- and thiocarbamates (54, 55), carbamates (56–58) and ureas (59–61) respectively (Fig. 7B).^17^ While strong alkoxide and thiolate nucleophiles reacted easily, weaker alcohol and amine nucleophiles required the addition of catalytic amounts of DMAP (4-dimethylaminopyridine) and a base. Carbamoyl azides 62 were accessed by reaction of 36 with sodium azide (Fig. 7C).^57^ Subsequent irradiation with blue LEDs in the presence of the photocatalyst [Ir(dtbbpy)(ppy)_2_]PF_6_ triggered the formation of nitrenes which cyclised to give N–CF_3_ (benz)imidazolidinones 63–65 (Fig. 7D).^58,59^ Nickel-catalysed alkynylation of *N*-trifluoromethyl carbamoyl fluorides 36 gave access to a range of alkyl and (hetero)aryl N–CF_3_ alkylamides 66 (Fig. 7E).^60^ Finally, reduction with NaBH_4_ or NaBD_4_ afforded *N*-trifluoromethylated formamides 67 and their deuterated isotopologues (Fig. 7F).^61^

Incorporation of a redox active ester in N–CF_3_ amide 69, derived from amino acid precursors, allowed for cyclisation under photoredox or electrochemical conditions to afford cyclic *N*-(trifluoromethyl)amides (Fig. 8).^62^ Handles for further functionalisation were incorporated on the arene, such halides (74), boronic esters (75), silanes (76) and amides. Functional groups that may be susceptible to oxidation (thioether 73) and radical addition (alkenes and alkynes 77) were tolerated under the reaction conditions. While electron-rich arenes gave good yields, no examples of electron-poor arenes were included in the scope, and only one heteroaromatic product (thiophene 79) was formed in low yields.

**Fig. 8:**
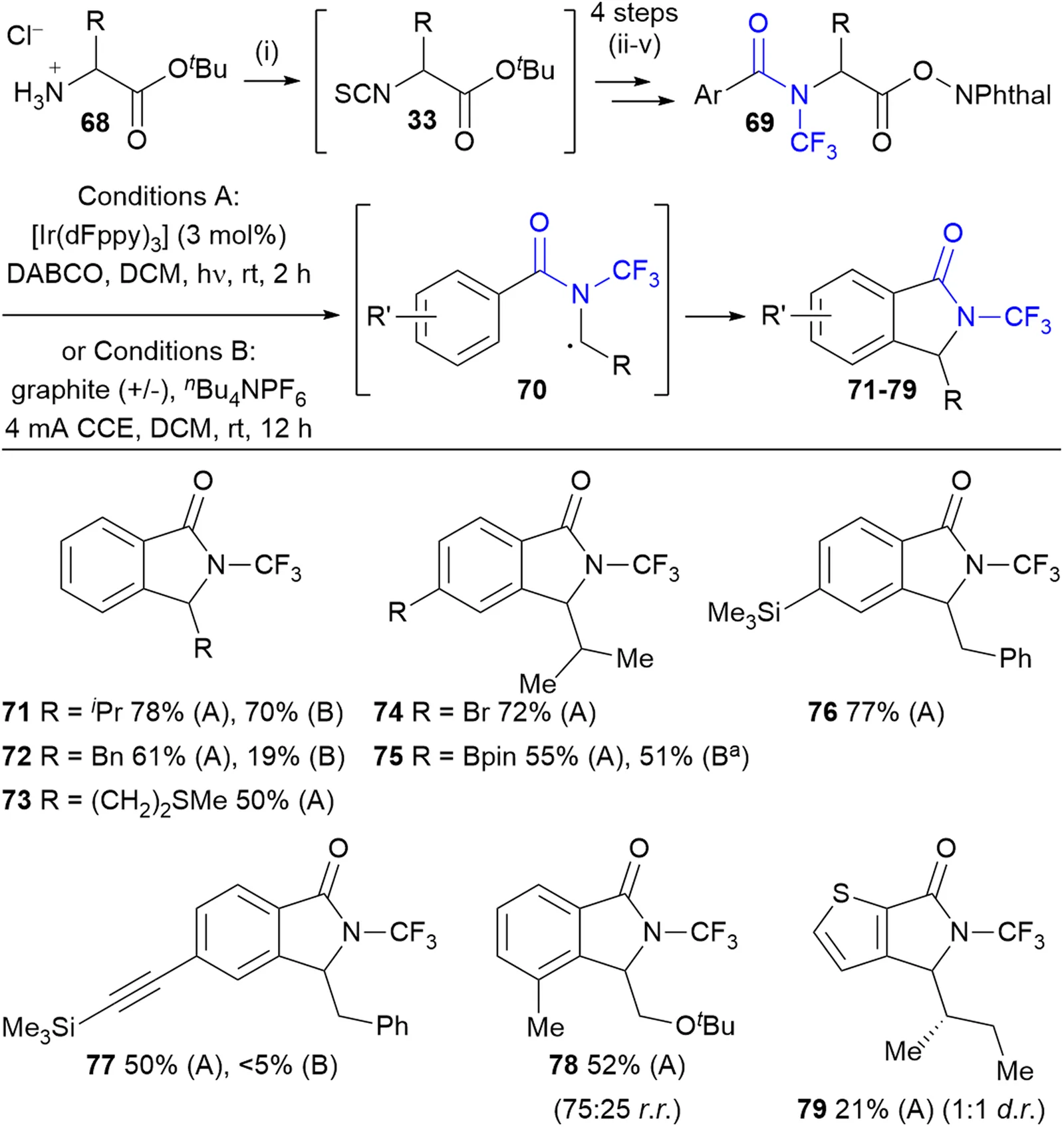
Synthesis of cyclic N–CF_3_ amides. (Yields are reported from 68.)^62^. Synthesis of 68: (i) triphosgene, NaHCO_3_ (aq), DCM, 0 °C, 1 h; (ii) AgF, BTC, MeCN, 50 °C, 45–83%; (iii) ArMgBr, THF, rt, 0.25–18 h, 37–98%; (iv) TFA, DCM, rt, 2 h; (v) DMAP, DIC, PhthN-OH, DCM, rt, 1 h, 23–94%. ^*a*^Modified electrochemical conditions: Ni-foam(−)/graphite(+), NiCl_2_·6H_2_O (5 mol%), dtbbpy, Bu_4_NPF_6_, NEt_3_, DMA, 1 mA CCE, rt, 4 h.

To avoid repeated handling of triphosgene, Schoenebeck *et al.* demonstrated that a stock solution of AgOCF_3_ could be used as a fluorophosgene reservoir for the synthesis of N–CF_3_ carbamoyl fluorides 36 (Scheme 3).^56^ Solutions of AgOCF_3_ in MeCN, prepared from triphosgene and AgF, were stable under inert conditions at −30 °C and released fluorophosgene upon activation with a nucleophile.

**Scheme 3:**
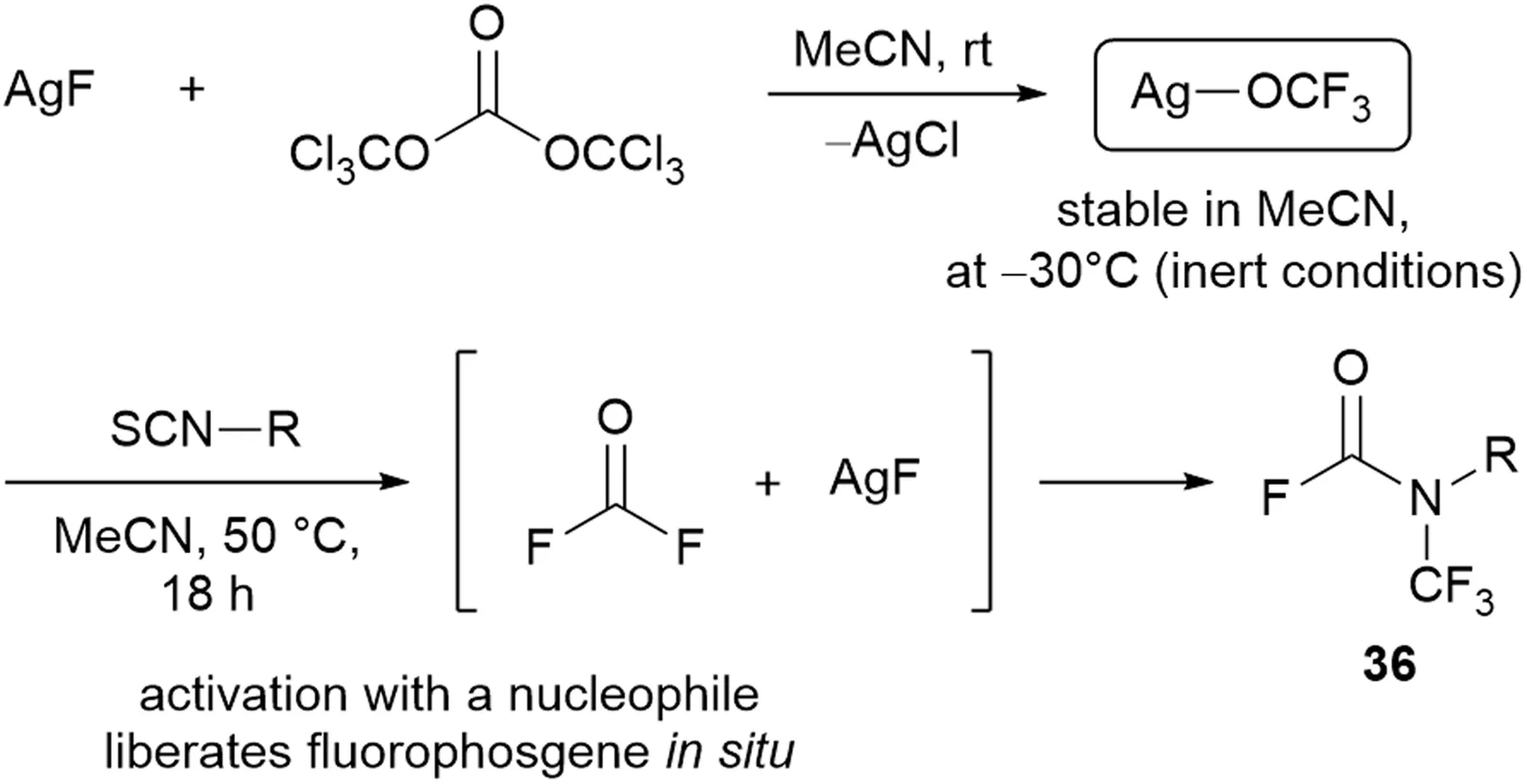
AgOCF_3_ as a fluorophosgene reservoir in the synthesis of N–CF_3_ carbamoyl fluorides.^56^

With the aim of synthesising pharmaceutically relevant N–CF_3_ derivatives, Chen *et al.* applied Schoenebeck's strategy to 4-pyridyl-isothiocyanates 33 derived from the corresponding phosphonium salts 80 (Fig. 9).^63^ A range of N–CF_3_ amides, (thio)carbamates and ureas were accessed. However, synthesis of the pyridyl-isothiocyanate starting materials 33 occurred *via* radical cation formation under oxidative conditions, requiring electron-poor pyridines and steric bulk at C3, and limiting the functional group compatibility of this method.

**Fig. 9:**
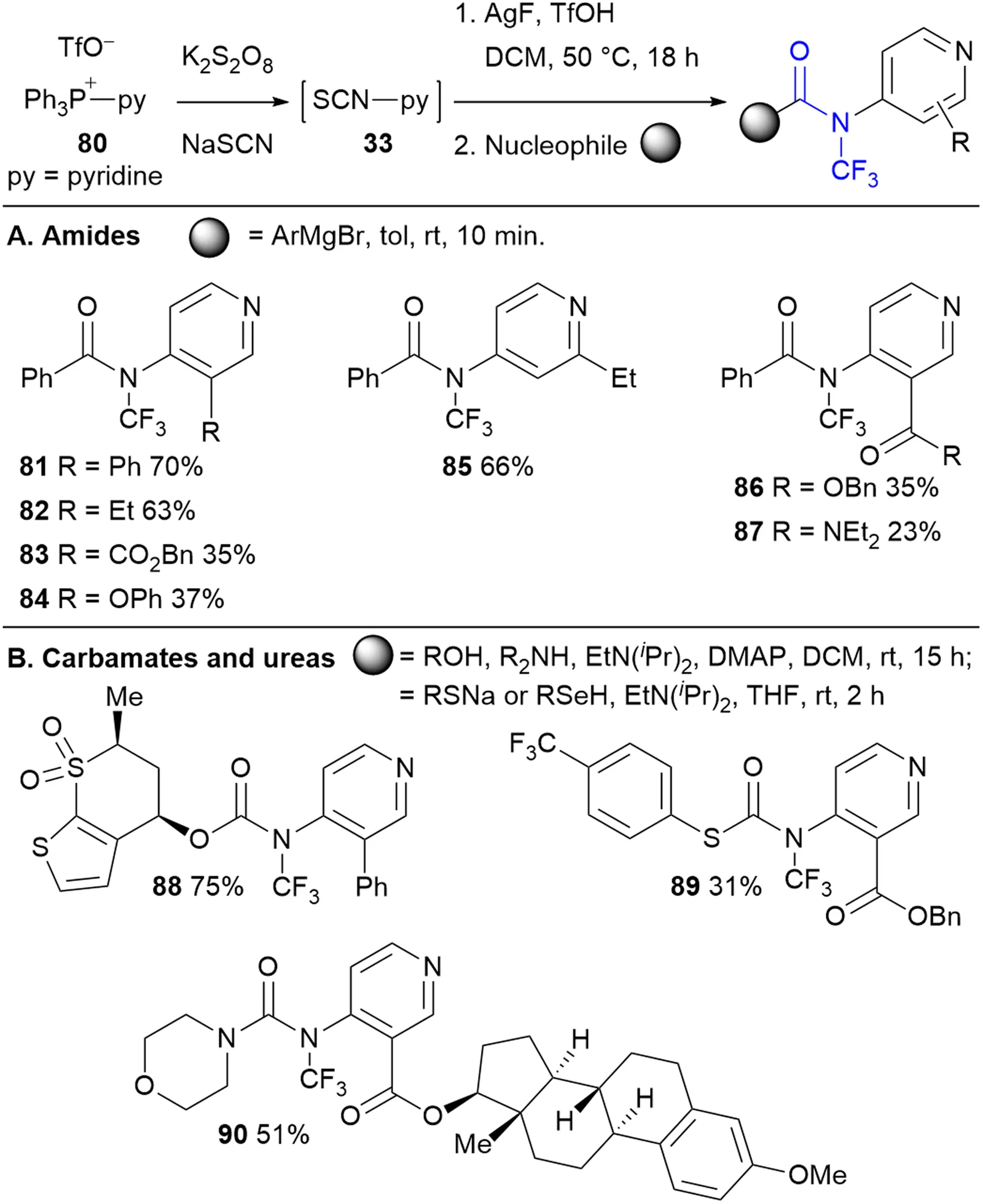
Synthesis of N–CF_3_ pyridyl amides from isothiocyanates. (Yields are reported over two steps from 80)^63^.

In 2021, Toste and Wilson recognised that the reactivity of the silver azanide anion 35 could be increased by activation with pyridine or 2,4,6-trimethylpyridine to allow for reaction with less reactive electrophiles such as acyl halides and pentafluorophenyl esters (Fig. 10).^52^ A comprehensive scope of alkyl, aryl and allyl isothiocyanates was reacted with aliphatic and (hetero)aromatic acyl halides carrying a range of functionalities including halides, alkenes, amides and esters. Heteroaromatics such as furan (99), thiophene (100), thiazole, pyridine (109, 110), pyrazine, pyrimidine (101), pyridazine, benzofuran, and quinoline did not impede the reaction, despite their potential to act as ligands for the azanide anion intermediate 35, like pyridine. Electron-deficient (hetero)aroyl chlorides gave better yields than electron-rich ones (96–102), presumably due to their increased electrophilicity, while electron-rich isothiocyanates resulted in more reactive azanide anions (104–107). Due to the lower reactivity of acyl halides, the scope of this transformation was more limited than that reported by Schoenebeck *et al.* (Fig. 7 above). Molecules with stereogenic centres (109) underwent racemisation, presumably due to deprotonation at the alpha carbon by the azanide anion 35. In some cases (*e.g.* the reaction of 4-iodophenyl isothiocyanate with benzoyl chloride), the azanide anion 35 reacted with the intermediate difluoromethylimine 34 instead of the acyl halide to afford carbamimidic fluoride 111. Sterics also had an important effect on the reaction: secondary isothiocyanates were incompatible with aliphatic acyl halides but gave reasonable yields with electron-deficient aroyl chlorides (108). Some of these limitations were addressed in a later expansion of the work.^64^ Optimisation of the AgF and pyridine loading led to higher yields with secondary—and even tertiary (110)—isothiocyanates, while simultaneously supressing racemisation (109).

**Fig. 10:**
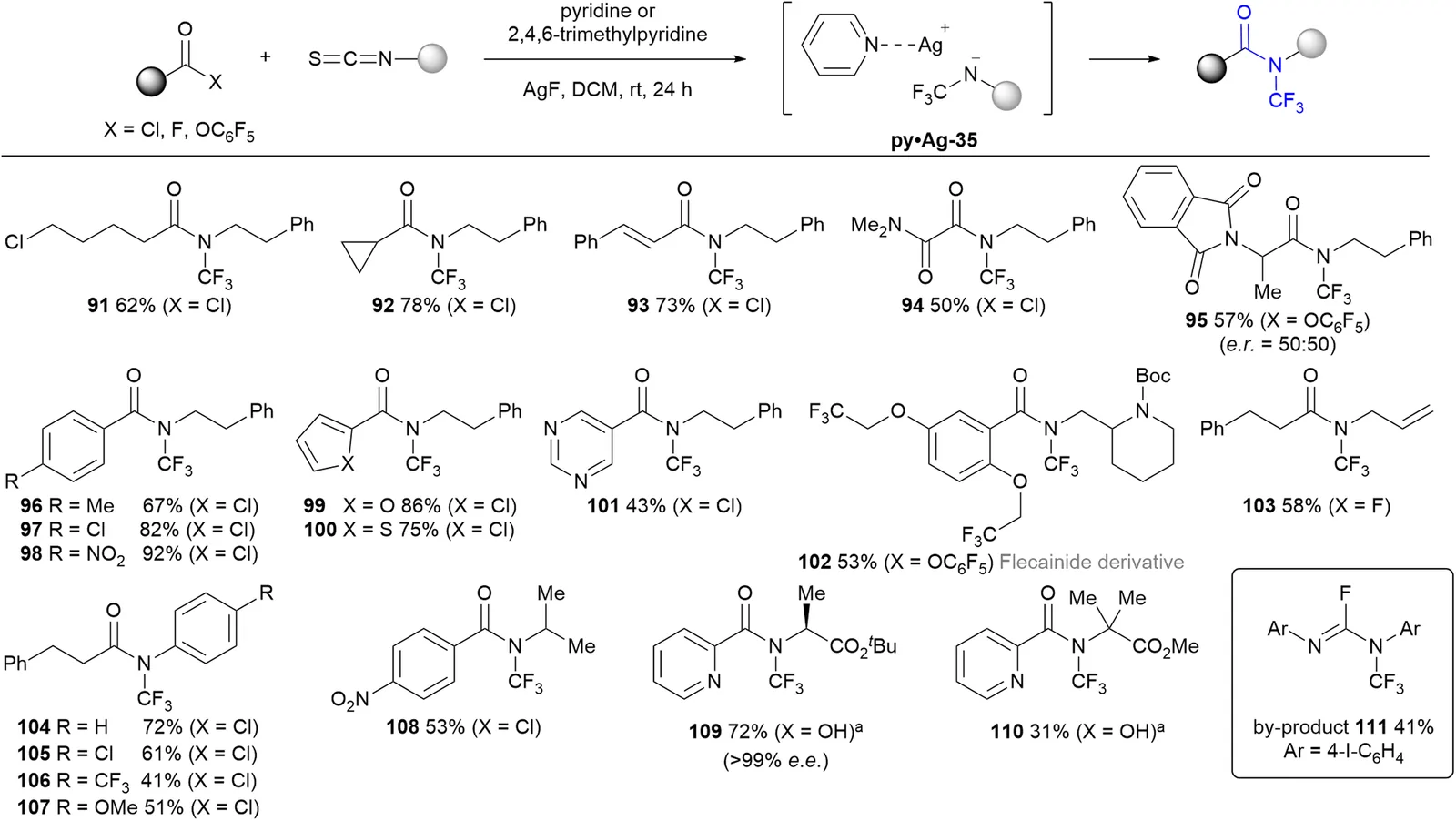
Synthesis of N–CF_3_ amides from isothiocyanates.^52^ (a) The acid chloride was made *in situ* from the carboxylic acid and Ghosez's reagent (1-chloro-*N*,*N*,2-trimethyl-propenylamine).^64^

### *O*-Protected *N*-(trifluoromethyl)(hydroxyl)amines

An early alternative to isothiocyanates as *N*-(trifluoromethyl)-amine precursors in amide coupling reactions were *O*-protected *N*-(trifluoromethyl)(hydroxyl)amines. Reaction of nitrosoarenes with Me_3_SiCF_3_ affords *O*-silylated *N*-(trifluoromethyl)(hydroxyl)amines in near quantitative yields. However, these products slowly decompose and are of limited synthetic use.^65^ In 2012, Inoue and Handa recognised that a protecting group change with acetic anhydride affords stable *O*-acylated *N*-(trifluoromethyl)(hydroxyl)amines 112 (Fig. 11).^50,51^ An alternative method for the synthesis of 112 with FC_3_SO_2_Na under copper catalysis was later reported by Selander and co-workers.^46^ Reduction of 112 with H_2_ and Pd/C forms *N*-(trifluoromethyl)amines 32, which can be trapped with anhydrides to yield the corresponding *N*-(trifluoromethyl)amides.^50^ The scope of the initial patent was limited to practically unfunctionalised *N*-(trifluoromethyl)(aryl)amines (113–118), which benefit from aromatic stabilisation, but it constituted an important proof of principle.

**Fig. 11:**
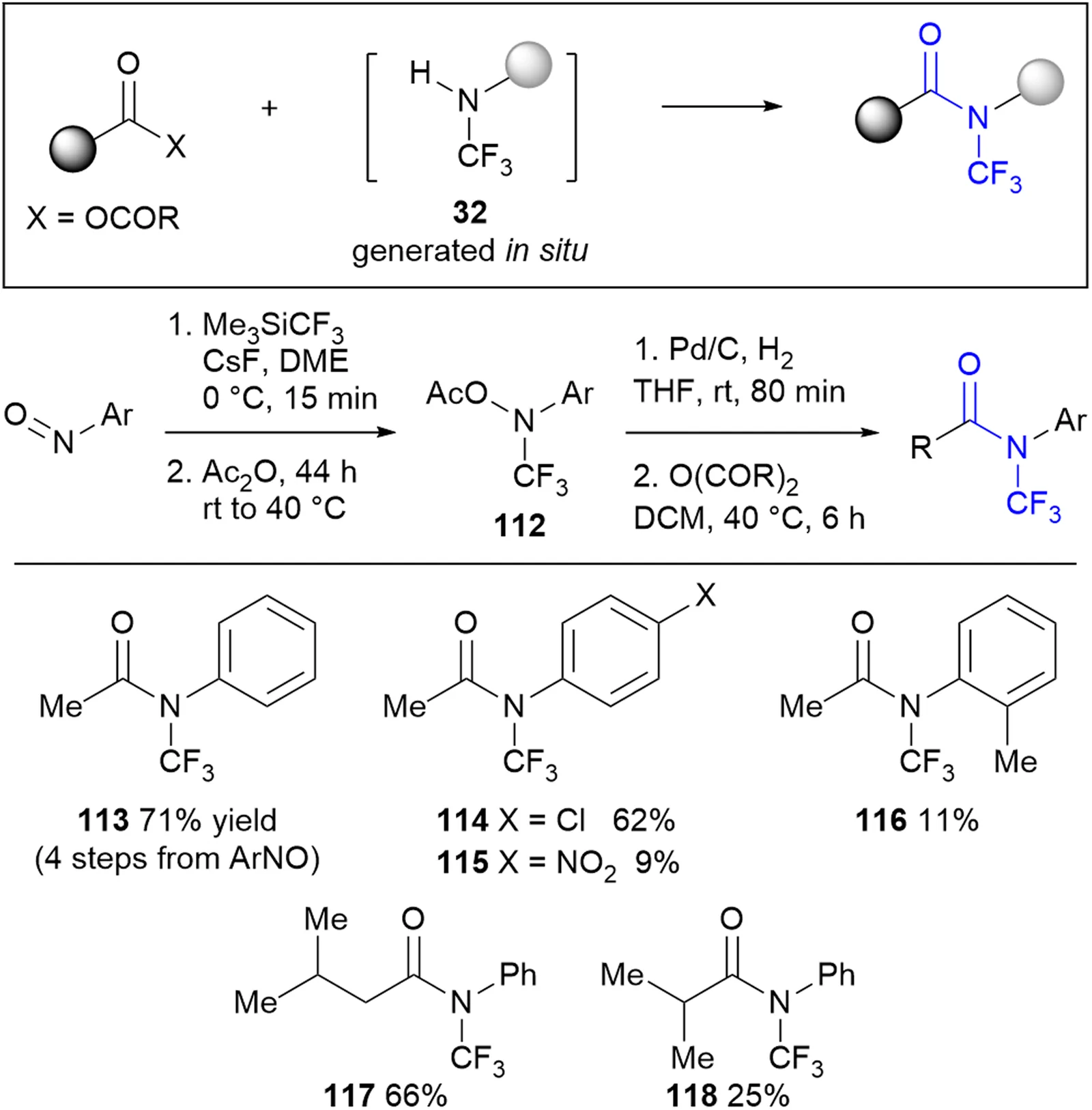
Amide coupling with *in situ* generated *N*-(trifluoromethyl)amines. (Yields are given over 4 steps from the corresponding nitrosoarene.)^50^

Xu and co-workers later expanded the method to *N*-(trifluoromethyl)(alkyl)amines, which are much less stable towards HF elimination, as well as *N*-(trifluoromethyl)(heteroaryl)amines (Fig. 12).^15^ Reaction of Cbz or Boc protected *O*-acyl hydroxylamines 119 with [AgCF_3_] afforded the corresponding *N*-trifluoromethylated *O*-acyl hydroxylamines (120, 121). These compounds underwent a range of further functionalisation: (a) C–H trifluoromethylamination of (hetero)-arenes under iridium photocatalysis to afford Cbz-protected *N*-(trifluoromethyl)(aryl)amines 122; (b) alkene di-functionalisation to afford Cbz-protected *N*-(trifluoromethyl)(alkyl)amines 123; and (c) trifluoromethylamination of styrenes with concomitant Boc hydrolysis and cyclisation to afford N–CF_3_ oxazolidinones 124. Reductive Cbz cleavage of 122 and 123 liberated *N*-(trifluoromethyl)amines 32, which were trapped *in situ* with phosgene and a range of nucleophiles (following Schoenebeck's conditions—see Fig. 7 above)^17^ to give *N*-trifluoromethylated amides (125–126, 130–133), carbamates (127), ureas (128), and carbamoyl azides (129, 134).

**Fig. 12:**
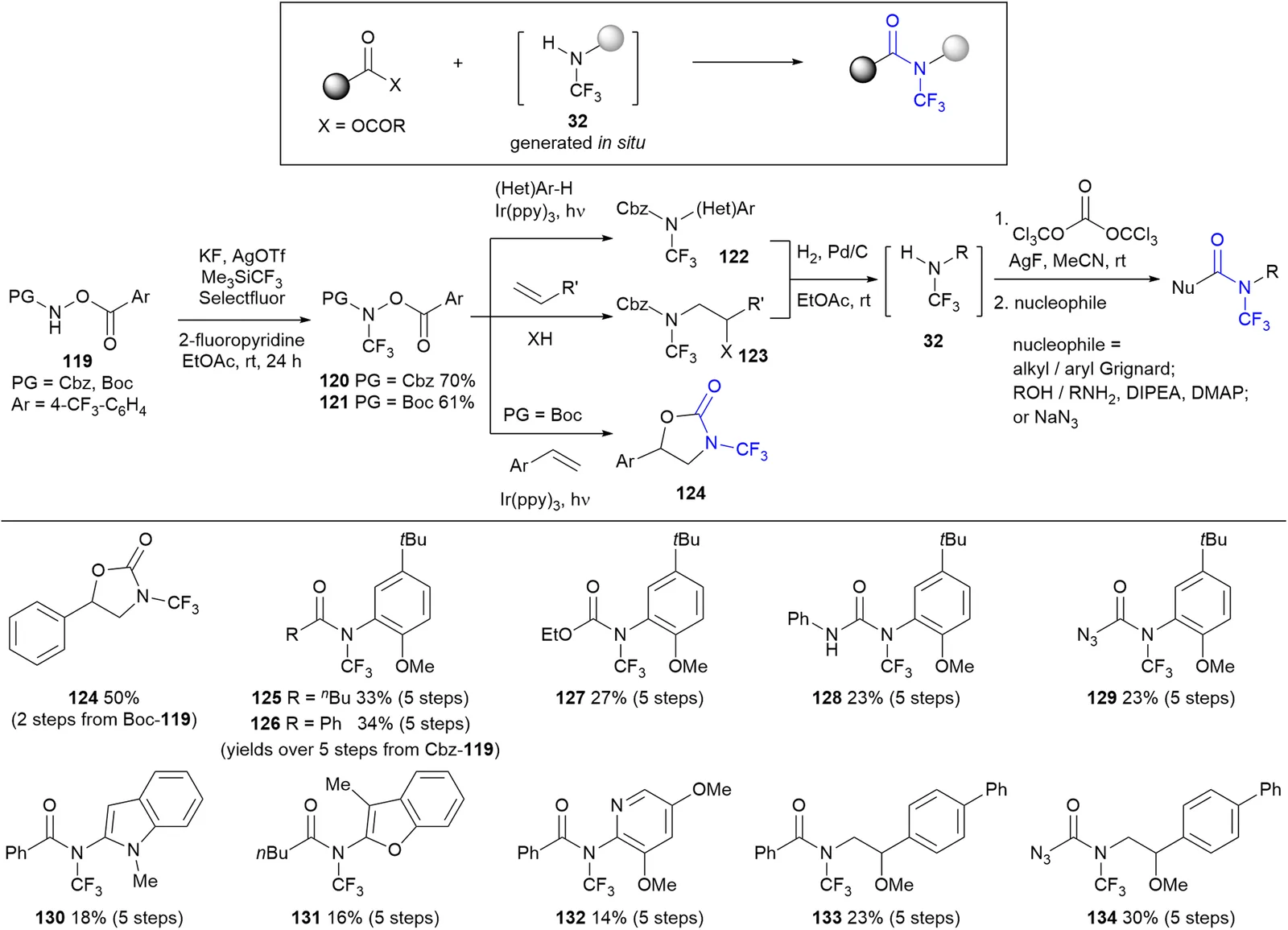
Amide coupling with *in situ* generated *N*-(trifluoromethyl)amines. (Yields are given over 2–5 steps from the corresponding hydroxylamine 119.)^15^

### Secondary *N*-(trifluoromethyl)amides

Compared with the tertiary amides discussed so far, methods for the synthesis of secondary *N*-(trifluoromethyl)amides are less well developed. Wenbin *et al.* reported the first synthesis of secondary N–CF_3_ amides by adapting Schoenebeck's methodology. AgF-mediated desulfurisation-fluorination of benzoyl isothiocyanates in the presence of Et_3_N·HF afforded secondary N–CF_3_ arylamides 135–137 (Fig. 13).^66^ The reported scope was extremely limited, and the stability of the products was not investigated, although they were reported to have been purified using silica column chromatography.

**Fig. 13:**
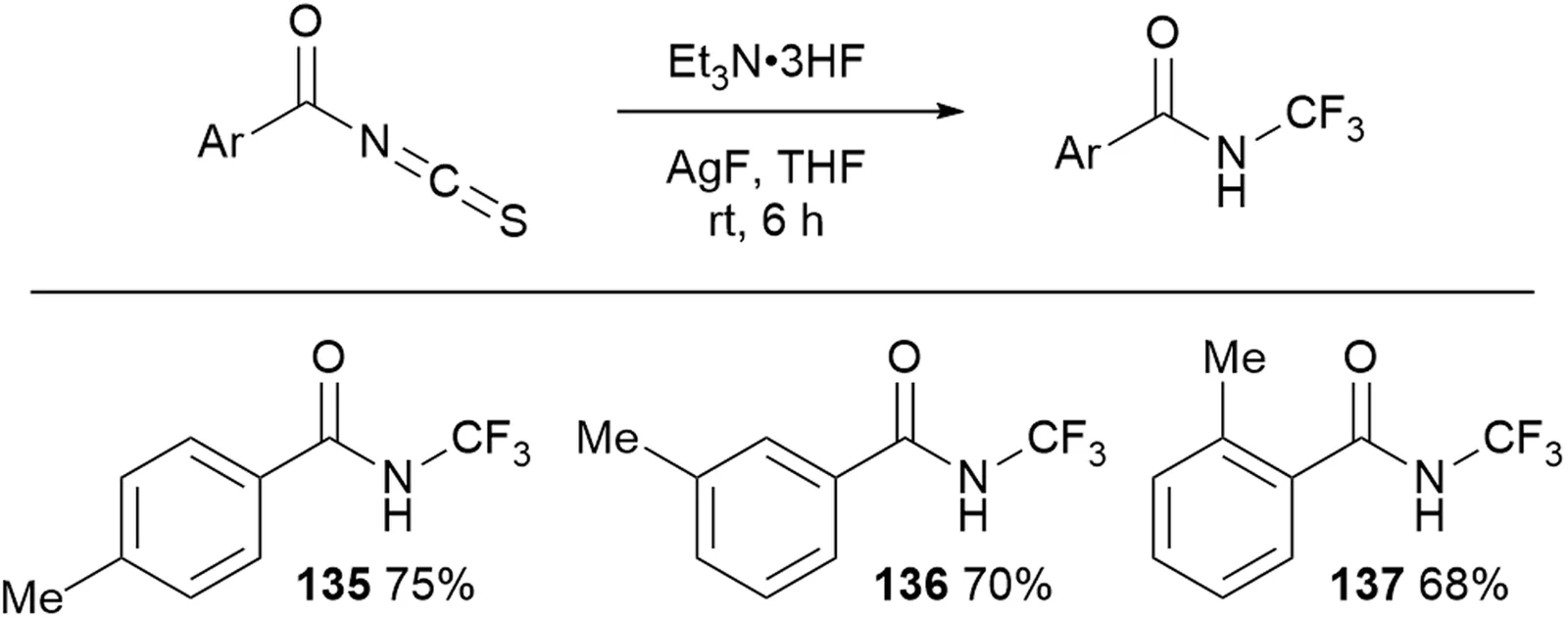
Synthesis of secondary N–CF_3_ amides from benzoyl isothiocyanates.^66^

In 2025, Beier *et al.* reported a complementary approach in which thermal degradation of N–CF_3_ 1,2,3-triazoles afforded ketenimines 138 which could be hydrolysed to the corresponding secondary N–CF_3_ alkylamides in the presence of water or aqueous HF (Fig. 14).^22^ The scope was relatively limited. Heteroaromatic (141, 143) and alkyl halide (145) functionality was tolerated, although alkyl iodide 146 was observed to rapidly oxidise under the reaction conditions. At the time of writing this review, Beier's publication was not yet peer-reviewed, but it constituted the most in-depth study of the properties and stability of secondary *N*-(trifluoromethyl)amides (discussed in the ‘Properties of *N*-(trifluoromethyl)amides’ section above).

**Fig. 14:**
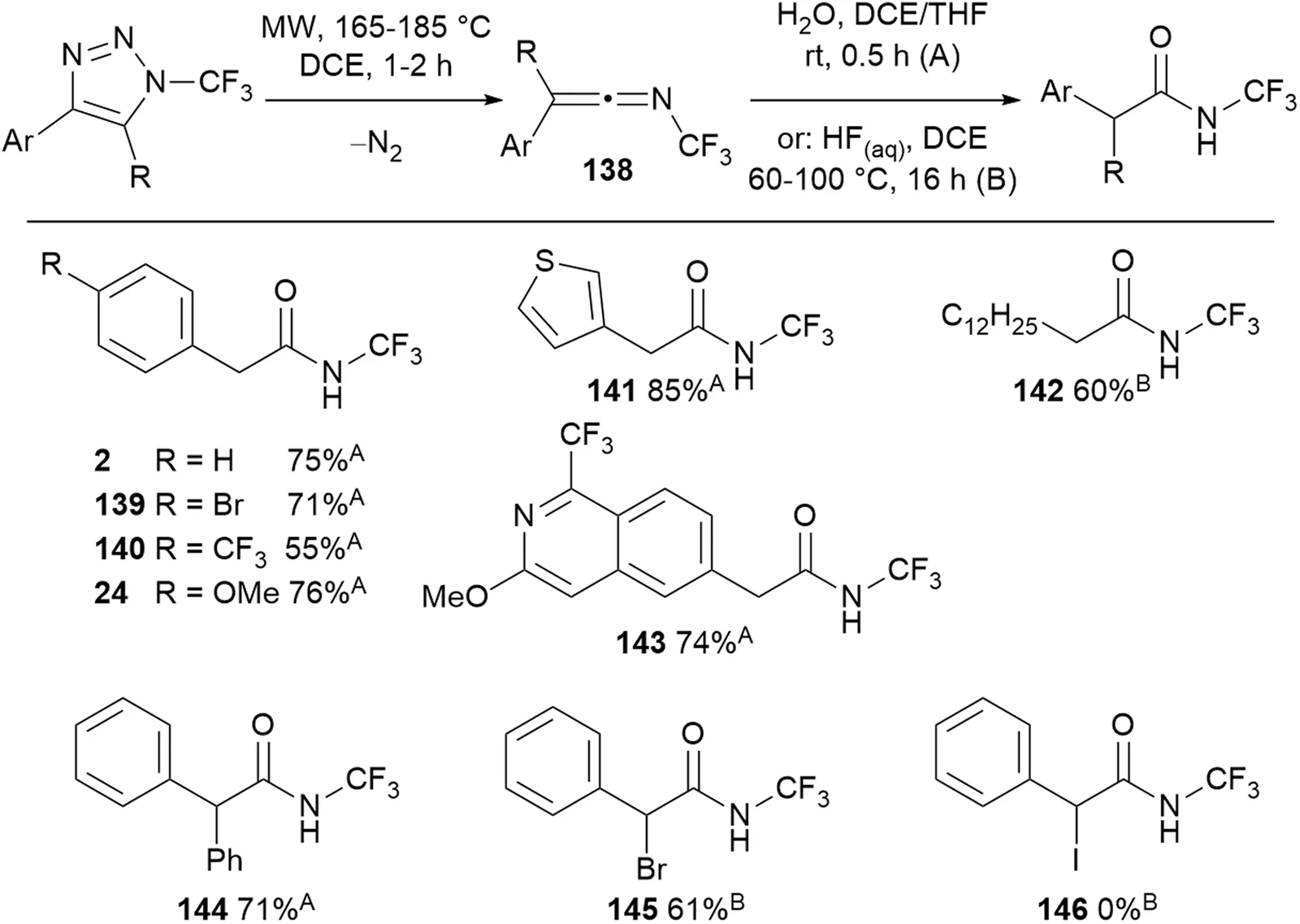
Synthesis of secondary N–CF_3_ amides from N–CF_3_ keteneimines.^22^

### Oxidative trifluoromethylation of amides

*N*-Methylation of amides can be achieved with electrophilic alkylating agents such as iodomethane.^67^ Attempts to achieve *N*-trifluoromethylation using a similar approach, however, are unsuccessful. This is likely due to the poor electrophilicity of ICF_3_ at lower temperatures (up to 80 °C) while higher temperatures (150 °C) lead to the decomposition of ICF_3_ to iodine and fluoride (Fig. 15).^68^

**Fig. 15:**
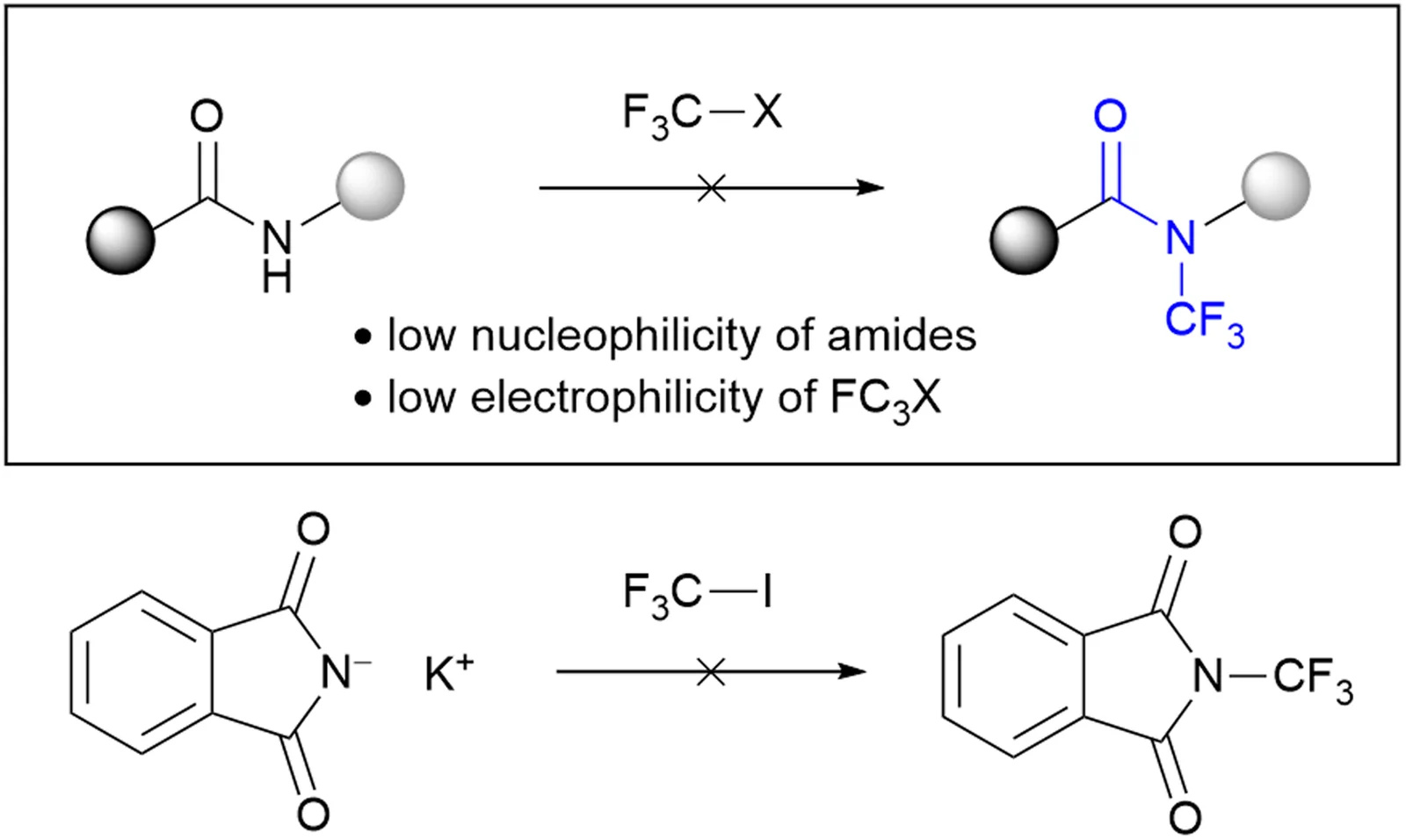
Trifluoromethylation of amides with CF_3_I.^68^

In 2020, Li *et al.* addressed this challenge with the formation of [AgCF_3_] 147 from AgOTf and Me_3_SiCF_3_ (Fig. 16).^69,70^ In the presence of Selectfluor, the initial Ag^I^CF_3_ species was oxidised to Ag^III^CF_3_, which underwent ligand exchange with deprotonated amides, resulting in amide-Ag^III^-CF_3_ complexes 148, from which the *N*-(trifluoromethyl)amide products were liberated through reductive elimination. The authors ruled out a radical mechanism, as incorporation of a cyclopropyl ring into the substrate did not result in the formation of any ring-opened products (155). Electron-rich and -poor *N*-(alkyl)aryl amides and *N*-(alkyl)alkyl amides were tolerated under the conditions (149–151), and a range of *N*-trifluoromethylated derivatives of complex molecules were synthesised in good yields (*e.g.* dehydrocholic acid derivative 156 and mexiletine derivative 157). Amino acid derivatives (158) and peptides were *N-*trifluoromethylated with retention of stereochemistry at the stereogenic centre. Heteroaromatic derivatives (159–160), *N*-(aryl)amides (161) and sterically demanding substrates (152, 154) resulted in reduced yields, with *N-tert*-butyl-substituted amides (154) failing to yield any products. Lactams (162) and carbamates (163) were also unsuccessful,^69^ but this limitation could be overcome by the addition of *O*-acyl and *O*-sulfonyl activating groups on the amide substrates (164, 165).^15^ The oxidative conditions required for this transformation make it fundamentally incompatible with oxidisable functional groups. However, Chu and co-workers demonstrated that an oxidant is unnecessary if the amide itself is pre-oxidised: reaction of *N*-bromo succinimide with AgF and Me_3_SiCF_3_ afforded high yields of *N*-(trifluoromethyl)succinimide 166 (Fig. 16), which was used as a reagent for the directed C–H trifluoromethylation of anilines.^71^

**Fig. 16:**
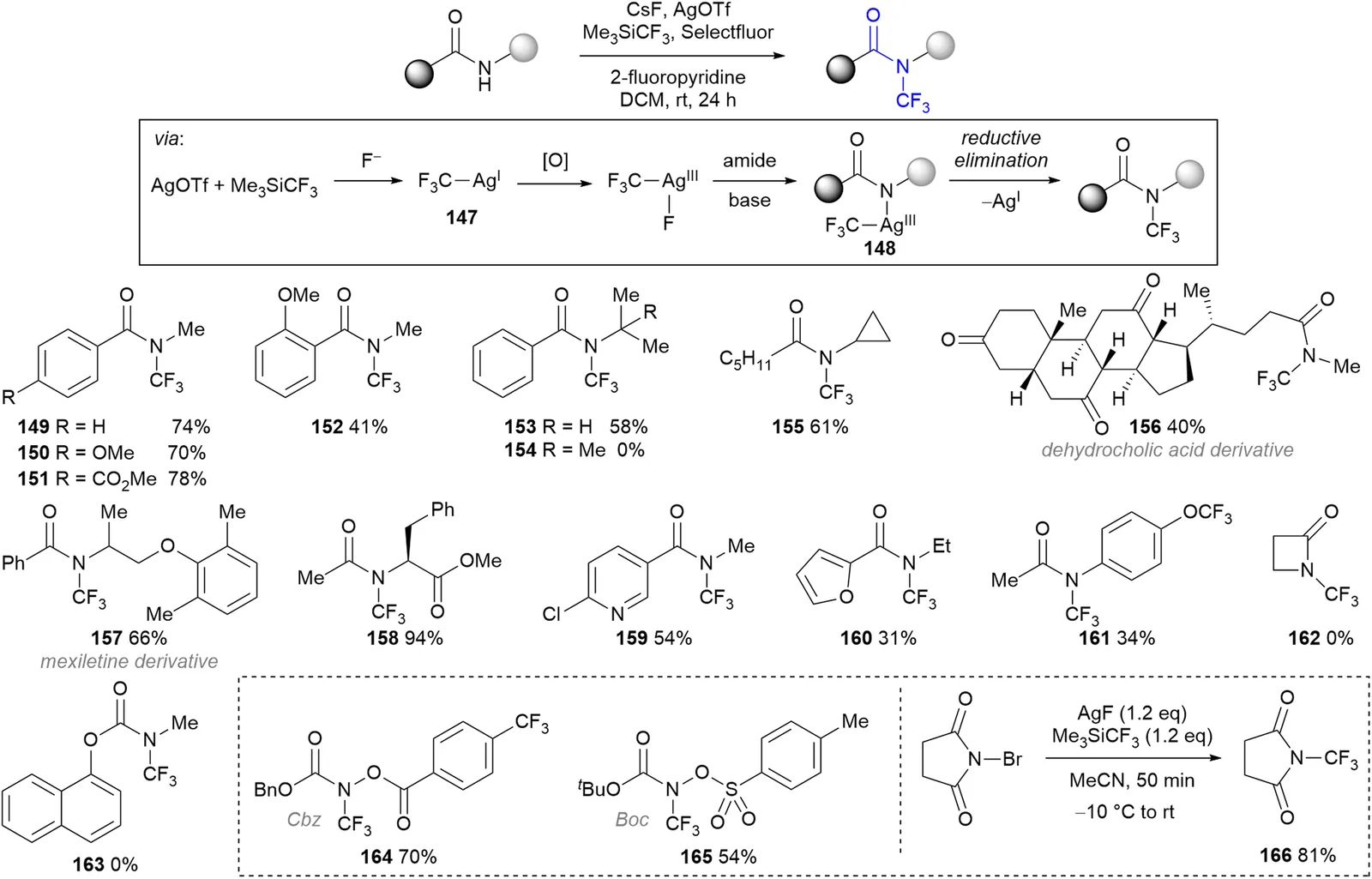
Oxidative trifluoromethylation of amides with [AgCF_3_].^15,69–71^

To address challenges associated with the oxidative conditions required in previous methods as well as the reliance on expensive silver salts, Lu and Shen recently developed a [Cu^I^CF_3_]-mediated synthesis of N–CF_3_-alkyl(aryl)amides (Fig. 17).^23^ Nitrenes, generated from 1,4,2-dioxazol-5-ones 167, were shown to insert into (CF_3_)_2_Cu(PPh_3_), affording N–CF_3_ copper amidates 168. These underwent substitution reactions with alkyl halides or Ullman-type coupling with aryldiazonium salts to yield N–CF_3_ amides.

**Fig. 17:**
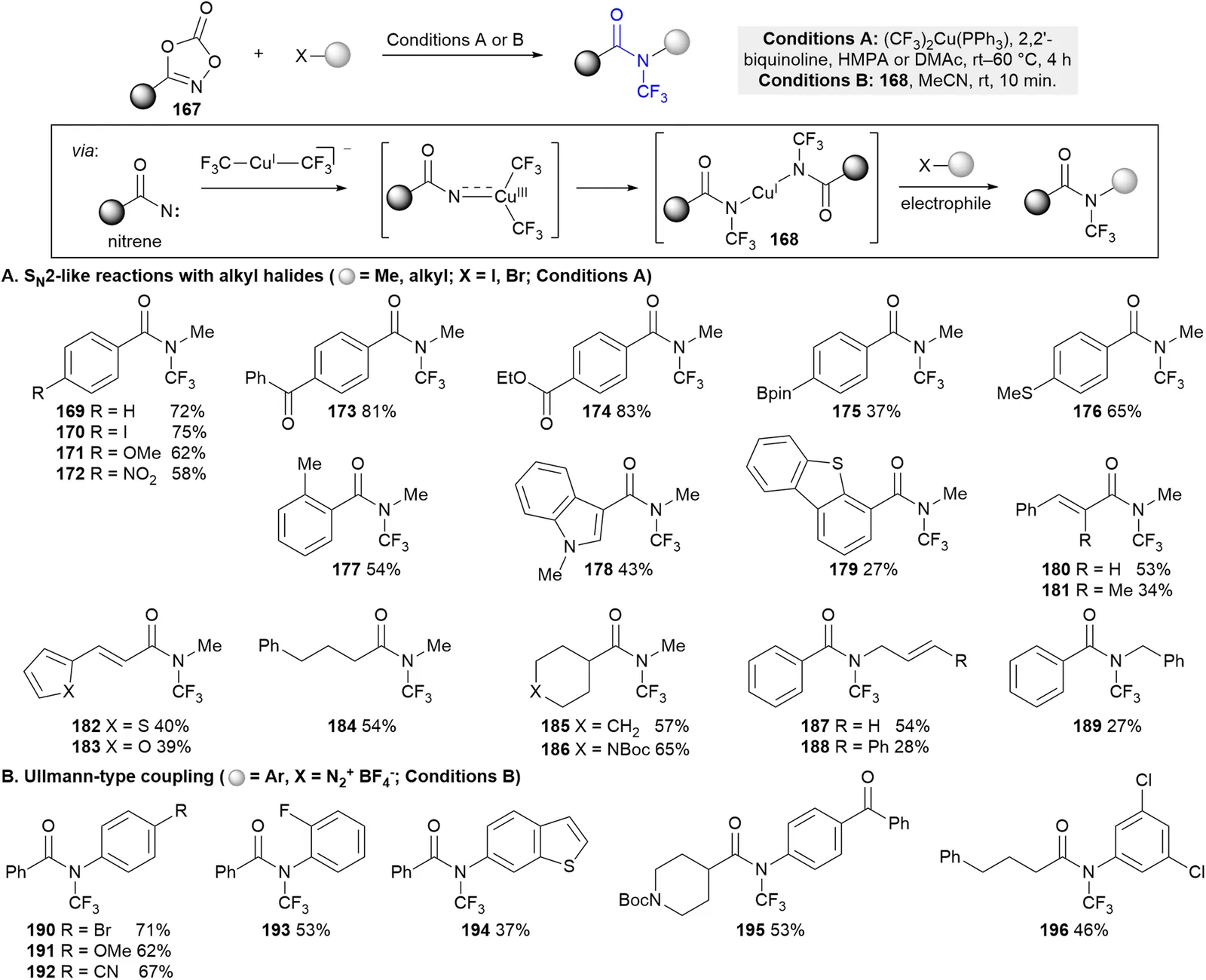
Copper-mediated N–CF_3_ amide synthesis *via* nitrene insertion into [Cu^I^–CF_3_].^23^

Reaction with iodomethane afforded a range of *N*-(methyl)amides (Fig. 17A). (Hetero)aryl, alkyl and alkenyl 1,4,2-dioxazol-5-ones 167 were competent nitrene precursors, incorporating these groups on the carbonyl moiety of the amide products (169–158). Functional groups that were incompatible with Grignard reagents, such as ketones (173), were tolerated in good yields. Oxidatively labile groups such as boronic esters (175), thioesters (176), alkenes (180–183) and alkynes, and heteroaromatics (178–179, 182–183) gave reduced yields, possibly due to competing oxidation by the nitrene intermediate. As with previous methods, steric bulk around the reactive centre also led to decreased yields (177, 181). Benzylic and tertiary nitrene precursors were incompatible with the reaction conditions, and protic functional groups required protection (178, 186). While reaction with the sterically unencumbered electrophile MeI generally proceeded in high yields, allyl and benzyl bromides afforded lower yields (187–189), which was attributed to competing side-reactions with (CF_3_)_2_Cu(PPh_3_) as well as the low nucleophilicity of the N–CF_3_ amide 168.

The isolated copper complex 168 was also shown to undergo Ullmann-type coupling reactions with (hetero)aryl diazonium salts affording *N*-(hetero)(aryl)amides, albeit in lower yields than the *N*-(alkyl)amides (Fig. 17B).

### Electrochemical C–H oxidation of tertiary *N*-methylamides

Direct C–H fluorination of *N*-(methyl)amides would present an atom-efficient route towards *N*-(trifluoromethyl)amides. Young *et al.* synthesised perfluoro-*N*,*N*-dimethylacetamide 198 from 2,2,2-trifluoro-*N*,*N*-dimethylacetamide 197 in 5% yield using this strategy (Scheme 4).^72^ However, the high solubility of the desired product 198 in the reaction solvent, HF, led to rapid decomposition to the corresponding acyl fluoride 199. Ingat'ev and co-workers hypothesised that less soluble perfluoro-*N*,*N-*dimethylacetamides would perform better, but yields remained moderate (≤33%).^73^

**Scheme 4:**
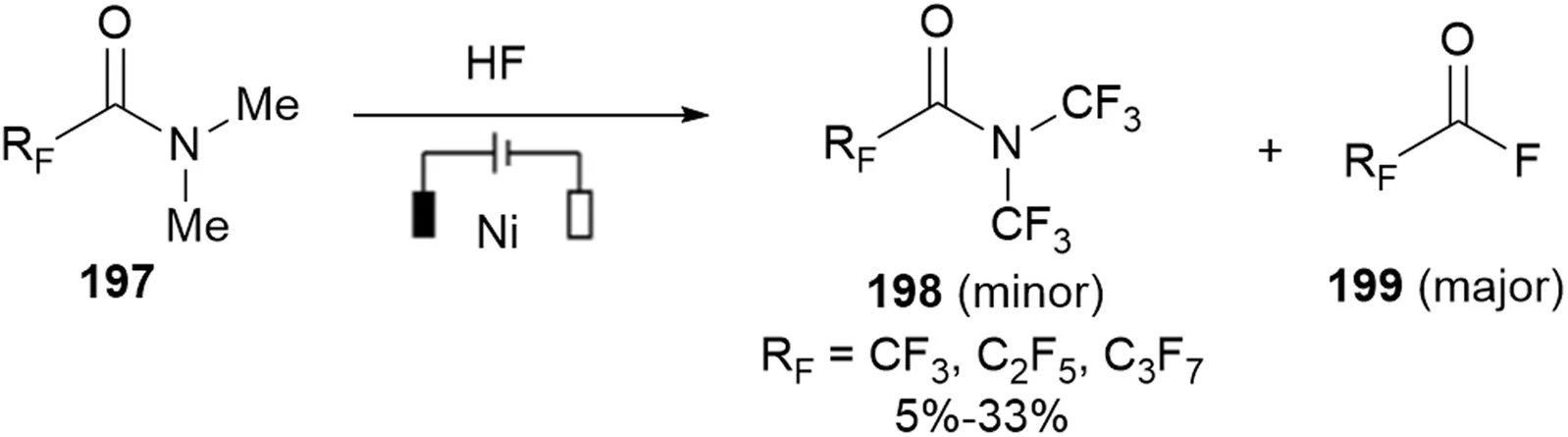
Electrochemical C–H oxidation of N–Me amides.^72,73^

### A radical strategy: *N*-(trifluoromethyl)amides from nitriles

Compared with N–CF_3_ azanide anions, N–CF_3_ radicals are more stable towards defluorination. Xu, Wang *et al.* exploited this fact in their synthesis of *N*-(trifluoromethyl)amides *via* N–CF_3_ amidyl radicals 204 (Fig. 18).^30^ Reaction of alkyl, vinyl and (hetero)aryl nitriles 200 with hypervalent iodine reagent 201 and pyridine *N*-oxides afforded radical precursors 203. Under iridium photocatalysis, 203 acted as a source of electrophilic N–CF_3_ amidyl radicals which could be trapped with a variety of electron-rich radical acceptors—including electron-rich (hetero)arenes—to afford *N*-(trifluoromethyl)amides in good yields (113, 205–215). Protection of protic groups was required (206*vs.*10) and electron-poor arenes resulted in sluggish reaction alongside a significant amount of *N*-(trifluoromethyl)-acetamide by product 216 caused by 204 undergoing hydrogen atom abstraction or reduction followed by protonation. The N–CF_3_ amidyl radical 204 also added to electron-rich alkenes, alkynes and isonitriles followed by radical cyclisation onto an adjacent aromatic ring, affording cyclic N–CF_3_ amides 217–221. Mono-, di- and trisubstituted alkenes performed well under these conditions. However, tetrasubstituted alkenes (219) did not afford any of the desired product, and styrenes underwent radical polymerisation under the reaction conditions. In the absence of adjacent arenes, the radical intermediate was also shown to cyclise onto adjacent carboxylic acids or amide groups (222), or quench through hydrogen atom abstraction (223). Reaction of 204 with allyl trimethylsilane afforded *N*-(allyl)-N–CF_3_ amide 224 and reaction with sily enol ethers allowed for the installation of ketone functionality *beta* to the amide (225).

**Fig. 18:**
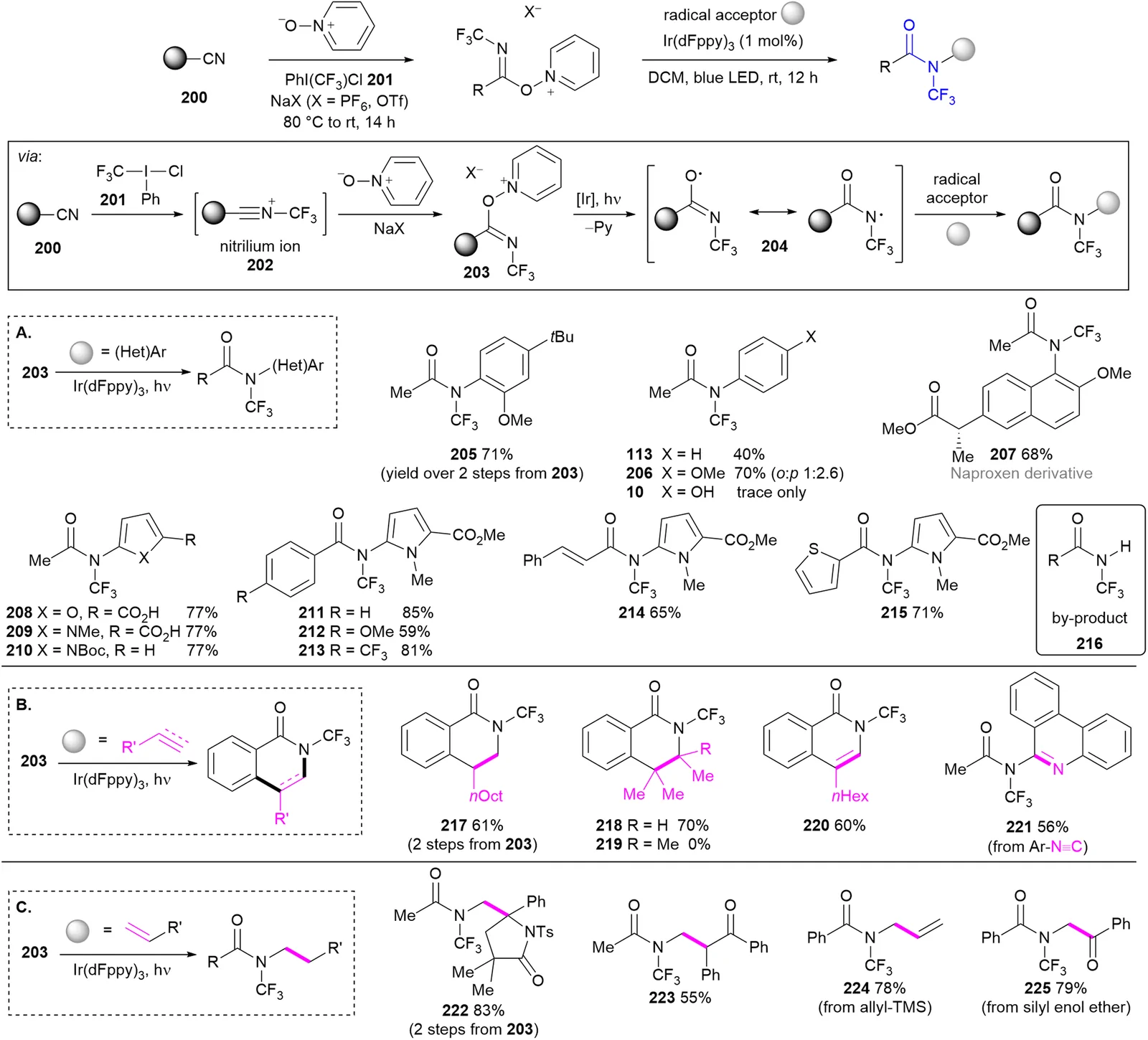
Synthesis of *N*-(trifluoromethyl)amides *via* a radical strategy. (Yields are given over 2 steps from the corresponding nitrile 200.)^30^

While this method presents a complementary strategy towards N–CF_3_ amides to those described above, the reactions require access to a glovebox and synthesis of the key hypervalent iodine reagent 201 is not straightforward, requiring careful temperature control (−40 °C for 24 h).^74^

## Conclusion

*N*-(Trifluoromethyl)amides represent an emerging class of functional groups in medicinal chemistry. Preliminary examination of their physical and pharmacokinetic properties suggest that they merge the benefits of amides and trifluoromethyl groups in drug design, and can impart beneficial features (by modulating p*K*_a_, lipophilicity and conformational rigidity). Their solubility and hydrolytic stability is generally sufficient for most pharmaceutical application, and can be further tailored through structural modifications.

While recent synthetic advances have made N–CF_3_ amides more accessible, challenges remain that pose promising avenues for future development:

• Due to the unique reactivity of fluorinated molecules, traditional strategies for amide synthesis cannot be translated 1-to-1 to the synthesis of N–CF_3_ amides. Some amine surrogates that have recently become popular in amide bond formation (isonitriles, isocyanates, sulfonamides, azides)^1^ are currently under-explored in N–CF_3_ amide synthesis.

• The main synthetic methods available to access *N*-(trifluoromethyl)amides rely on oxidative generation of [MCF_3_] complexes or the reaction of N–CF_3_ carbamoyl fluorides with Grignard reagents, limiting functional group compatibility. Stereogenic centres are often racemised and steric bulk around the reactive centre reduces yields substantially. Cyclic N–CF_3_ amides are underrepresented in current scopes, as are secondary *N*-(trifluoromethyl)amides.

• Direct C–H fluorination of N–Me amides under electrochemical conditions would offer a highly atom-economic route towards N–CF_3_ amides, but methods so far remain under-developed and low yielding.

• Strategies using N–CF_3_ radicals circumvent stability issues associated with N–CF_3_ amines. The only method using this strategy (so far) relies on the use of a hypervalent iodine reagent that, itself, is not straightforward to synthesise.

• Further studies into the metabolic stability of trifluoromethylamides—especially secondary N–CF_3_ amides—are required if they are to evolve their full potential as bioactive compounds.

## Author contributions

MOD: conceptualisation, writing – original draft; ILW, IB, CMcI: writing – review & editing.

## Conflicts of interest

There are no conflicts to declare.

## Data Availability

No primary research results, software or code have been included and no new data were generated or analysed as part of this review.
